# Supplementary figures and images for: Using a low-dose ultraviolet-B lighting solution during working hours: An explorative investigation towards the effectivity in maintaining healthy vitamin D levels
Source: PLoS One. 2023 Mar 31;18(3):e0283176. doi: 10.1371/journal.pone.0283176 (PMC10065255; doi:10.1371/journal.pone.0283176)

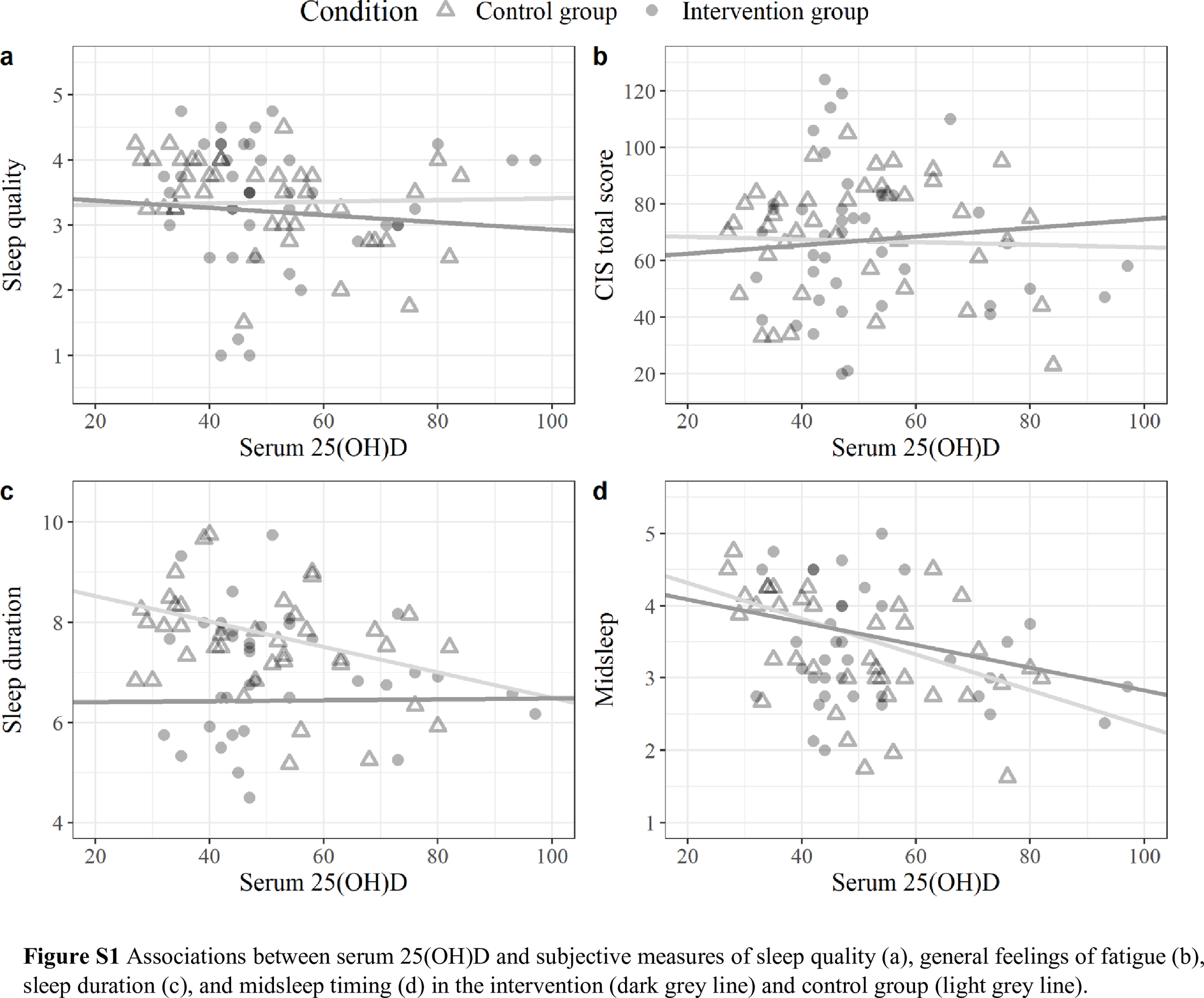

Supplement: S1 Fig — (TIF) [file pone.0283176.s002.tif]
