# Supplementary material for: Using a low-dose ultraviolet-B lighting solution during working hours: An explorative investigation towards the effectivity in maintaining healthy vitamin D levels
Source: PLoS One. 2023 Mar 31;18(3):e0283176. doi: 10.1371/journal.pone.0283176 (PMC10065255; doi:10.1371/journal.pone.0283176)
Supplement: S1 Table — (PDF) [file pone.0283176.s003.pdf]

Table S1 Parameter estimates linear mixed model analysis; relationship between serum 25(OH)D and sleep quality in the control group and intervention group

**Fixed effects (control group)**	**Estimate (unstandardized coefficient)**	***SE***	**95% CI**	***t*-value**	***p*-value**
Intercept	3.29	0.46	2.35 – 4.22	7.10	<0.001
Vitamin D	0.001	0.01	-0.01 – 0.02	0.17	0.87
Measurement:					
Week 1 vs. Week 4	0.10	0.15	-0.20 – 0.40	0.70	0.49
Week 1 vs. Week 8	0.01	0.15	-0.30 – 0.33	0.10	0.92
**Random effects (control group)**	**Estimate**	***SE***	**95% CI**	***z***	***p*-value**
Level 2 Intercept	0.35	0.17	0.13 – 0.93	2.00	0.05
Level 1 Residual	0.11	0.03	0.06 – 0.19	3.42	0.001

**Fixed effects (intervention group)**	**Estimate (unstandardized coefficient)**	***SE***	**95% CI**	***t*-value**	***p*-value**
Intercept	3.49	0.55	2.38 – 4.60	6.37	<0.001
Vitamin D	-0.01	0.01	-0.02 – 0.01	-0.67	0.51
Measurement:					
Week 1 vs. Week 4	0.36	0.15	0.05 – 0.67	2.37	0.03
Week 1 vs. Week 8	0.17	0.18	-0.18 – 0.53	1.00	0.33
**Random effects (intervention group)**	**Estimate**	***SE***	**95% CI**	***z***	***p*-value**
Level 2 Intercept	0.72	0.29	0.32 – 1.58	2.48	0.01
Level 1 Residual	0.12	0.03	0.07 – 0.21	3.73	<0.001
