# Supplementary material for: Using a low-dose ultraviolet-B lighting solution during working hours: An explorative investigation towards the effectivity in maintaining healthy vitamin D levels
Source: PLoS One. 2023 Mar 31;18(3):e0283176. doi: 10.1371/journal.pone.0283176 (PMC10065255; doi:10.1371/journal.pone.0283176)
Supplement: S2 Table — (PDF) [file pone.0283176.s004.pdf]

Table S2 Parameter estimates linear mixed model analysis; relationship between serum 25(OH)D and sleep duration in the control group and intervention group

**Fixed effects (control group)**	**Estimate (unstandardized coefficient)**	***SE***	**95% CI**	***t*-value**	***p*-value**
Intercept	9.03	0.76	7.46 – 10.61	11.81	<0.001
Vitamin D	-0.03	0.01	-0.05 – 0.001	-1.99	0.06
Measurement:					
Week 1 vs. Week 4	-0.12	0.30	-0.73 – 0.48	-0.41	0.68
Week 1 vs. Week 8	-0.28	0.32	-0.93 – 0.37	-0.88	0.38
**Random effects (control group)**	**Estimate**	***SE***	**95% CI**	***z***	***p*-value**
Level 2 Intercept	0.44	0.24	0.14 – 1.31	1.78	0.08
Level 1 Residual	0.50	0.14	0.28 – 0.87	3.50	<0.001

**Fixed effects (intervention group)**	**Estimate (unstandardized coefficient)**	***SE***	**95% CI**	***t*-value**	***p*-value**
Intercept	6.39	0.90	4.55 – 8.22	7.07	<0.001
Vitamin D	0.001	0.01	-0.03 – 0.03	0.95	0.95
Measurement:					
Week 1 vs. Week 4	0.83	0.32	0.17 – 1.48	2.57	0.02
Week 1 vs. Week 8	0.67	0.35	-0.05 – 1.39	1.87	0.07
**Random effects (intervention group)**	**Estimate**	***SE***	**95% CI**	***z***	***p*-value**
Level 2 Intercept	0.82	0.42	0.30 – 2.22	1.97	0.05
Level 1 Residual	0.51	0.15	0.29 – 0.91	3.41	0.001
