# Supplementary material for: Using a low-dose ultraviolet-B lighting solution during working hours: An explorative investigation towards the effectivity in maintaining healthy vitamin D levels
Source: PLoS One. 2023 Mar 31;18(3):e0283176. doi: 10.1371/journal.pone.0283176 (PMC10065255; doi:10.1371/journal.pone.0283176)
Supplement: S3 Table — (PDF) [file pone.0283176.s005.pdf]

Table S3 Parameter estimates linear mixed model analysis; relationship between serum 25(OH)D and general fatigue in the control group and intervention group

**Fixed effects (control group)**	**Estimate (unstandardized coefficient)**	***SE***	**95% CI**	***t*-value**	***p*-value**
Intercept	69.30	14.91	39.00 – 99.61	4.65	<0.001
Vitamin D	-0.05	0.24	-0.54 – 0.441	-0.19	0.85
Measurement:					
Week 1 vs. Week 4	4.64	5.39	-6.30 – 15.57	0.86	0.40
Week 1 vs. Week 8	2.09	5.53	-9.12 – 13.29	0.38	0.71
**Random effects (control group)**	**Estimate**	***SE***	**95% CI**	***z***	***p*-value**
Level 2 Intercept	238.15	110.54	95.88 – 591.51	2.15	0.03
Level 1 Residual	154.58	41.35	91.51 – 261.11	3.74	<0.001

**Fixed effects (intervention group)**	**Estimate (unstandardized coefficient)**	***SE***	**95% CI**	***t*-value**	***p*-value**
Intercept	59.34	16.81	25.30 – 93.38	3.53	0.001
Vitamin D	0.15	0.26	-0.38 – 0.03	0.58	0.57
Measurement:					
Week 1 vs. Week 4	-0.72	5.00	-10.91 – 9.47	-0.14	0.89
Week 1 vs. Week 8	1.14	5.68	-10.40 – 12.67	0.20	0.84
**Random effects (intervention group)**	**Estimate**	***SE***	**95% CI**	***z***	***p*-value**
Level 2 Intercept	498.04	211.36	80.41 – 232.27	2.36	0.02
Level 1 Residual	136.66	36.98	216.78 – 1144.22	3.70	<0.001
