# Supplementary material for: Using a low-dose ultraviolet-B lighting solution during working hours: An explorative investigation towards the effectivity in maintaining healthy vitamin D levels
Source: PLoS One. 2023 Mar 31;18(3):e0283176. doi: 10.1371/journal.pone.0283176 (PMC10065255; doi:10.1371/journal.pone.0283176)
Supplement: S4 Table — (PDF) [file pone.0283176.s006.pdf]

Table S4 Parameter estimates linear mixed model analysis; relationship between serum 25(OH)D and midsleep in the control group and intervention group

**Fixed effects (control group)**	**Estimate (unstandardized coefficient)**	***SE***	**95% CI**	***t*-value**	***p*-value**
Intercept	4.81	0.49	3.78 – 5.83	9.77	<0.001
Vitamin D	-0.02	0.01	-0.04 – -0.01	-3.03	0.01
Measurement:					
Week 1 vs. Week 4	-0.21	0.25	-0.72 – 0.30	-0.83	0.42
Week 1 vs. Week 8	-0.40	0.26	-0.93 – 0.13	-1.53	0.14
**Random effects (control group)**	**Estimate**	***SE***	**95% CI**	***z***	***p*-value**
Level 2 Intercept	0.07	0.09	0.01 – 0.76	0.84	0.40
Level 1 Residual	0.38	0.11	0.21 – 0.66	3.56	<0.001

**Fixed effects (intervention group)**	**Estimate (unstandardized coefficient)**	***SE***	**95% CI**	***t*-value**	***p*-value**
Intercept	4.40	0.59	3.18 – 5.62	7.45	<0.001
Vitamin D	-0.02	0.01	-0.04 – 0.003	-1.68	0.11
Measurement:					
Week 1 vs. Week 4	-0.47	0.27	-1.03 – 0.09	-1.72	0.10
Week 1 vs. Week 8	0.01	0.26	-0.51 – 0.54	0.06	0.96
**Random effects (intervention group)**	**Estimate**	***SE***	**95% CI**	***z***	***p*-value**
Level 2 Intercept	0.17	0.13	0.03 – 0.79	1.25	0.21
Level 1 Residual	0.36	0.11	0.20 – 0.66	3.31	0.001
