# Supplementary material for: Using a low-dose ultraviolet-B lighting solution during working hours: An explorative investigation towards the effectivity in maintaining healthy vitamin D levels
Source: PLoS One. 2023 Mar 31;18(3):e0283176. doi: 10.1371/journal.pone.0283176 (PMC10065255; doi:10.1371/journal.pone.0283176)
Supplement: S1 File — (PDF) [file pone.0283176.s007.pdf]

RESEARCH PROTOCOL

Explorative investigation towards the effectivity of a special lighting solution in influencing 25(OH)D serum levels

(June 2020)

PROTOCOL TITLE 'Explorative investigation towards the effectivity of a special lighting solution in influencing 25(OH)D serum levels'

Protocol ID	NL75389.015.20 - W20.022
Short title	Vitamin D3 pilot
EudraCT number	Not applicable
Version	2.0
Date	29-06-2020
Coordinating investigator/project leader	
Principal investigator(s) :<br><br><br><br><br>Investigators:	Prof. Yvonne de Kort<br>Human Technology Interaction group<br>Eindhoven University of Technology,<br>P.O. Box 513, 5600 MB Eindhoven,<br>The Netherlands,<br><br>Dr. Laura Huiberts<br>Human Technology Interaction group<br>Eindhoven University of Technology,<br>P.O. Box 513, 5600 MB Eindhoven,<br>The Netherlands,<br><br>Dr. Karin Smolders<br>Human Technology Interaction group<br>Eindhoven University of Technology,<br>P.O. Box 513, 5600 MB Eindhoven,<br>The Netherlands,
Sponsor (in verrichter/opdrachtgever)	Dr. B.M.I. van der Zande<br>Signify Netherlands B.V.<br>High Tech Campus 7<br>5656 AE Eindhoven<br>The Netherlands
Subsidising party	Signify
Independent expert (s)	Prof. dr. S. Overeem<br>Biomedical Diagnostic Group<br>Dept of Electrical Engineering

	Eindhoven University of Technology,<br>P.O. Box 513, 5600 MB Eindhoven,<br>The Netherlands,
Laboratory sites <if applicable>	Diagnostiek Voor U<br>Contactpersoon: Simone Pouwels<br>Boschdijk 1119, 5626 AG Eindhoven
Pharmacy <if applicable>	

PROTOCOL SIGNATURE SHEET

Name	Signature	Date
Head of Department Human-Technology interaction, Eindhoven University of Technology<br><br>Chris Snijders		29-06-2020
Coordinating Investigator/Project leader/Principal investigator<br><br>Prof. Yvonne de Kort<br>Human Technology Interaction group<br>Eindhoven University of Technology,<br>P.O. Box 513, 5600 MB Eindhoven,<br>The Netherlands,		29-06-2020
Sponsor<br>Dr. B.M.I. van der Zande<br>Signify Netherlands B.V.<br>High Tech Campus 7<br>5656 AE Eindhoven<br>The Netherlands		06102020

TABLE OF CONTENTS

NL72938.015.20 - W20.022 2
1. INTRODUCTION AND RATIONALE 9
2. OBJECTIVES 11
2.1 Primary objective 11
2.2 Secondary Objectives 11
3. STUDY DESIGN 12
3.1 Duration of the study 13
3.2 Setting of the study 13
4. STUDY POPULATION 14
4.1 Population base 14
4.2 Inclusion criteria 14
4.3 Exclusion criteria 14
4.4 Sample size calculation 14
5. TREATMENT OF SUBJECTS 18
6. INVESTIGATIONAL PRODUCT 19
6.1 Technical description 19
6.2 Operating behavior 21
7. METHODS 23
7.1 Study parameters/endpoints 23
7.1.1 Main study parameter/endpoint 23
7.1.2 Secondary study parameters/endpoints (if applicable) 23
7.1.3 Other study parameters (if applicable) 23
7.2 Randomisation, blinding and treatment allocation 24
7.3 Data treatment 25
7.3.1 General 25
7.3.2 Data treatment of inclusion/exclusion criteria 26
7.3.3 Main study parameter: Vitamin D3 serum levels 27
7.3.4 Secondary parameters on sleep and general fatigue 27
7.4 Study procedures 28
7.4.1 The data assessment weeks 29
7.4.1.1 Blood sampling to measure 25OHD serum levels 29
7.4.1.2. Questionnaires 30
7.4.1.3 UV-dose measurements 30
7.5 Withdrawal of individual subjects 32
7.6 Premature termination of the study 32
8. SAFETY REPORTING 32
8.1 Temporary halt for reasons of subject safety 32
8.2 AEs, SAEs and SUSARs 32

8.2.1 Adverse events (AEs) 32
8.2.2 Serious adverse events (SAEs) 33
8.2.3 Suspected unexpected serious adverse reactions (SUSARs) 33
8.3 Annual safety report 34
8.4 Follow-up of adverse events 34
8.5 Data Safety Monitoring Board (DSMB) / Safety Committee 34
9. STATISTICAL ANALYSIS 35
10. ETHICAL CONSIDERATIONS 36
10.1 Regulation statement 36
10.2 Recruitment and consent 36
10.3 Objection by minors or incapacitated subjects (if applicable) 37
10.4 Benefits and risks assessment, group relatedness 37
10.4.1 Benefits 37
10.4.2 Possible burden 38
10.4.3 Potential risk 38
10.5 Incentives (if applicable) 39
11. ADMINISTRATIVE ASPECTS, MONITORING AND PUBLICATION 40
11.1 Handling and storage of the data and documents 40
11.2 Monitoring and Quality Assurance 40
11.3 Amendments 41
11.4 Annual progress report 41
11.5 Temporary halt and (prematurely) end of study report 41
11.6 Public disclosure and publication policy 41
12. STRUCTURED RISK ANALYSIS 42
12.1 Identified risks 42
12.1.1 Mechanical, thermal and electrical 42
12.1.2 Photobiological safety 44
12.2 Application precautions 46
12.3 Risk - Benefit Analysis 47
12.4 CE-mark 48
13. REFERENCES 49

LIST OF ABBREVIATIONS AND RELEVANT DEFINITIONS

SED	Standard Erythemal dose: A standardized measure of erythemogenic UV radiation. One SED is equivalent to an erythemally effective radiant exposure of 100 J/m2 when the radiation is weighted by the erythema action spectrum normalized to its maximum, ser(λ).
UVI	The ultraviolet **index** or **UV Index** is an international standard measurement of the strength of sunburn-producing ultraviolet (**UV**) radiation at a particular place and time.
UVB	Ultra violet (UV) radiation, UVB specifically refers to the wavelength regime 295–320 nm, required for vitamin D3 production
LED	Light emitting diode
ipRGC	Intrinsic photosensitive retinal ganglion cell
D3	Refers to vitamin D3: cholecalciferol (made from 7-dehydrocholesterol in the skin). In 1935, the chemical structure of vitamin D3 was established and proven to result from the ultraviolet irradiation of 7-dehydrocholesterol.
PIA	Privacy Interest Assessment
FFS	Free floor standing (a luminaire type)
BSA	body surface area
25(OH)D	alcifediol, also known as calcidiol, 25-hydroxycholecalciferol, or 25-hydroxyvitamin D3 (abbreviated 25(OH)D), is a precursor of vitamin D3 (cholecalciferol) that is produced in the liver by hydroxylation by the enzyme cholecalciferol 25-hydroxylase. Physicians worldwide measure this metabolite to determine a patient's vitamin D3 status.
AE	Adverse Event
AR	Adverse Reaction
CA	Competent Authority
CCMO	Central Committee on Research Involving Human Subjects; in Dutch: Centrale Commissie Mensgebonden Onderzoek
CV	Curriculum Vitae
DSMB<br>EMA	Data Safety Monitoring Board<br>Ecological Momentary Assessment
EU	European Union
EudraCT<br>FACT	European drug regulatory affairs Clinical Trials<br>Flexible Assertive Community Treatment
GCP	Good Clinical Practice
IB	Investigator’s Brochure
IC	Informed Consent
IMP	Investigational Medicinal Product
IMPD	Investigational Medicinal Product Dossier

ISWT	Instructions for Sleep-Wake Timing
METC	Medical research ethics committee (MREC); in Dutch: medisch ethische toetsing commissie (METC)
(S)AE	(Serious) Adverse Event
SPC	Summary of Product Characteristics (in Dutch: officiële productinfomatie IB1-tekst)
Sponsor	The sponsor is the party that commissions the organisation or performance of the research, for example a pharmaceutical company, academic hospital, scientific organisation or investigator. A party that provides funding for a study but does not commission it is not regarded as the sponsor, but referred to as a subsidising party.
SUSAR	Suspected Unexpected Serious Adverse Reaction
Wbp	Personal Data Protection Act (in Dutch: Wet Bescherming Persoonsgevens)
WMO	Medical Research Involving Human Subjects Act (in Dutch: Wet Medisch-wetenschappelijk Onderzoek met Mensen

SUMMARY

Rationale: Vitamin D3 is essential for people's general health. Sunlight, through its UVB irradiance, delivers the most efficient route to sufficient vitamin D3 levels. Unfortunately, the modern lifestyles in urban and often indoor environments lead to a lack of UV exposure in the natural way. Moreover, it is not possible to create sufficient vitamin D in the skin during the autumn and winter months, while vitamin D intake via food is not sufficient. Therefore, supplementation with artificial UVB light seems an unobtrusive solution. This study explores whether addition of artificial low and safe doses of UVB to LED lighting solutions is sufficient to maintain healthy vitamin D3 levels for office workers. We hypothesize that the participants exposed to ultra-low doses of UVB during office hours will demonstrate a less rapid decline in 25(OH)D levels than the control group without UVB supplementation.

Objective: The key objective of the pilot study is to test the effectiveness of a desk-based lighting intervention to sustain 25(OH)D levels of office workers during autumn/winter months. The secondary objective is to explore the correlations between changes in serum 25(OH)D and indicators of sleep timing, sleep quality and general levels of fatigue.

Study design: This 8-week pilot study involves 28 home office workers in which type of lighting is manipulated between subjects (low-dose UVB light exposure vs. no artificial UVB exposure). Block randomization will be used to allocate participants to these conditions. Vitamin D blood levels and participants' self-reports on fatigue and sleep timing and quality be conducted three times, with 4-week intervals, supplemented with extremely short daily diaries (i.e., desk presence, time spent outside and body exposure surface).

Study population: Participants are healthy office workers, aged 18 and older, living in or around Eindhoven, working in their home office for at least 2.5 days a week. Participants should not be taking any oral vitamin D3 or use sunbeds (or planning to). Their skin should be of Type II or III (Fitzpatrick skin type). Participants can further not participate if they are breast feeding, pregnant (or planning to become pregnant), have (had) any malignant skin conditions, use any photosensitive medical drugs or have photosensitive medical conditions.

Intervention (if applicable): The individual home office workspaces of 14 office workers will be equipped with the desk-based intervention light and subsequently be exposed to a low dose of artificial UVB during daytime hours with a maximum of 8 hours and 20 min (<0.5 SED, UVI =~0.06 under normal application conditions). The other 14 office workers will act as the control group.

Main study parameters/endpoints: Changes in serum 25(OH)D serum levels.

Nature and extent of the burden and risks associated with participation, benefit and group relatedness: The main burden for participation in the study will be the discomfort experienced during blood sampling and the time consumption associated with the blood sampling and completing the questionnaires on sleep timing and quality, and general fatigue (~ 10 min. per long questionnaire and less than 1 minute per daily diary probing desk presence and time spent outside). The frequency of the blood sampling and completing the long questionnaire is three times during eight weeks with an estimated time consumption of 10 minutes per questionnaire and 10 minutes for each blood sampling. Risk assessment in relation to the ultra-low doses UVB indicated that the potential risk associated with photobiological safety are mitigated to negligible/tolerable residual risk.

1. INTRODUCTION AND RATIONALE

In the current 24-hour society, we spend about 90% of our time indoors [1]. Due to this, many of us do not receive the quality and quantity of light exposure that is essential for optimal daytime functioning as well as a good night's sleep. Several studies have already shown that sufficient light exposure during daytime is needed for good sleep quality as well as daytime functioning in terms of cognitive performance, mood, and vitality [2,3]. These effects of light exposure on wellbeing are also called the non-image forming (NIF) effects of light as they follow a different pathway than the image-forming effects [4,5,6]. The NIF effects of light are established via light-sensitive retinal photoreceptors which project to the biological clock (which resides in the suprachiasmatic nucleus) and several brain areas related to sleep, alertness, and cognition [5]. The sensitivity curve of the ipRGC was found to peak around 480 nm [7,8,9].

In addition to the importance of enough daytime exposure to visible light, the non-visible components of light, like UV exposure, have demonstrated to regulate bodily mechanisms as well [10, 11]. The varied effects derive, in part, from the differences among UV-A (320-400 nm), UVB (280-320 nm) and UV-C (100-280 nm) photons energy. UV photons are absorbed by UV sensitive molecules in skin and initiate a variety of photochemical reactions resulting in structural changes that could lead to positive or negative biological effects depending on the UV dose. Negative effects are associated to the formation of cyclobutylpyrimidine dimers in DNA contributing to the mechanism of erythema and sunburn formation [12].

A positive biological effect, initiated by the UVB sensitive chromophore in the epidermis, is 7-dehydrocholesterol (7-DHC, provitamin D3). UVB photons penetrating into human skin are responsible for its conversion into the active form of vitamin D3. The most well-known function of vitamin D3 is its role in the development of strong bones and prevention of rickets [13]. However, the vitamin D3 hormone is a very important and versatile molecule with a much larger impact on the human body than bone strength alone: it is also involved in a very large number of genes in the human genome, and hence a large number of tissues and cells. The last years, more and more attention is payed to vitamin D3 deficiency and the possible correlations with a wide number of health issues, like the immune system, autism, ADHD, coronary heart diseases, diabetes, several cancers, neuropsychiatric diseases (Alzheimer, dementia), infectious diseases, respiratory tract infections (influenza), cognitive functioning, and autoimmune diseases [11,14,15], where the vitamin D3 active metabolite 1,25(OH)2D3, is recognized as a critical hormone regulating cell growth and modulating the immune system [16].
Interestingly, sufficient vitamin D3 levels also seem to be essential for optimal day-to-day functioning via its influence on serotonin production during daytime, as well as its regulating effects on melatonin production during the evening and nighttime [17]. Via this modulating role on serotonin and melatonin, it is also expected that vitamin D deficiency may play a role in promoting a good sleep and general feelings of vitality and fatigue during the day. Indeed, several studies have already revealed significant

associations between serum 25(OH)D (the common vitamin D metabolite measured in the blood) and sleep in the general healthy population, where higher daytime serum 25(OH)D was significantly related to better sleep quality and a longer sleep duration [18-21]. However, intervention studies investigating the causal role of increasing vitamin D status (e.g. through supplementation) on sleep indicators are still lacking. With respect to fatigue, intervention studies have revealed that chronic fatigue symptoms significantly improve after correcting a vitamin D deficiency in people with chronic illness [22] as well as healthy people [23].

Despite the importance of vitamin D3 on people's health, a global pandemic in vitamin D3 insufficiency is occurring in the general healthy population [24-26]. Worldwide, between 60-90% of the population has insufficient serum 25(OH)D3 levels (<74 nmol/L) [27]. During the winter period at relatively higher latitudes, it is impossible to gain sufficient vitamin D synthesis through UVB exposure due to the large zenith angle of the sun. The same is true for times earlier than 10:00 or later than 15:00 in spring or summer [28]. The contribution of dietary vitamin D is generally too low to maintain sufficient serum 25(OH)D levels in healthy people [29,30]. Furthermore, it is important to note that vitamin D synthesis via UVB exposure is much more efficient, since the half-life value of serum 25(OH)D is much longer from UVB (sunlight) exposure, than from supplements or fortification, due to the regulatory processes in the skin [31]. Therefore, supplements should be taken very regularly to have high and stable serum 25(OH)D levels throughout the year. The Vitamin D3 absorption from supplements differs widely from person to person and depends also on several co-factors (e.g., level of fat tissue where vitamin D is stored). To this end, adherence to a vitamin D supplementation scheme is very important, which may not be easy for everyone. Finally, UVB exposure also exclusively leads to the synthesis of different vitamin D metabolites in the body (not synthesized with supplementation) that likely have important contributions to health and wellbeing, such as anti-inflammatory effects [32].

Because of the above mentioned benefits of UVB exposure to synthesize vitamin D, supplementation with artificial UVB light seems an unobtrusive solution to increase vitamin D status that could give advantages over alternative options. Previous published studies with UV light sources (controlled and uncontrolled, single centered studies) suggest that exposure to very low intensity narrow band UVB (i.e. significantly less UV than monthly ambient doses from sunlight) as well as low body surface area exposure may be adequate for maintenance of normal healthy vitamin levels throughout winter [33-38]. However, the available intervention studies generally investigated relative short exposure times (minutes to one hour max per session), using sunbeds with a full body exposure in participants characterized by a vitamin D insufficiency/deficiency executed in the winter months.

Therefore, we would like to explore whether low and safe doses of UVB exposure applied in office lighting solutions is sufficient to maintain healthy vitamin D3 levels for office workers. The main aim of this pilot study is to gain insights in the extent to which the intervention light with ultra low (<0.5 SED, UVI =~0.06) and safe (according to the IEC62471) UVB doses is able to sustain serum vitamin D3 levels (i.e., 25(OH)D) in healthy office workers (working from home) performing their usual job routines

and wearing their usual clothing. Additionally, we would like to explore correlations between changes in serum 25(OH)D levels and sleep (quality and duration) and general levels of fatigue during the day since vitamin D has been found to play a role in serotonin and melatonin regulation [17]. Based upon the results of the current pilot study, a larger and longer follow-up study with the aim to test whether office workers' health, sleep and feelings of fatigue are also impacted by improving serum vitamin D levels is envisioned.

2. OBJECTIVES

2.1 Primary objective

The primary objective of the proposed pilot study is to gain insight in the effectiveness of a desk-based lighting device with white light containing ultra-low and safe (according to the IEC62471) doses of artificial UVB irradiation in influencing serum 25(OH)D in the Fall/Winter months. Therefore, an ultra-low and safe daily artificial UVB dose is applied during workdays in the home office over a period of eight weeks (eight hours a day) which leads to the primary research question: **To what extent is an ultra-low (<0.5SED, UVI = ~0.06) and safe daily artificial UVB dose, evenly applied during workdays in a home office over a period of eight weeks (eight hours a day) during autumn, able to sustain the summer serum 25(OH)D levels in healthy office workers?**

We hypothesize that the participants exposed to ultra-low doses of UVB during office hours (while working at home in the home office) will demonstrate a less rapid decline in 25(OH)D levels during the eight-week study period than the control group without UVB supplementation.

2.2 Secondary Objectives

As mentioned in the introduction, sufficient 25(OH)D levels also appear essential for optimal daytime functioning (i.e. feelings of energy/vitality) and sleep via its influence on serotonin production during daytime, as well as its influence on melatonin production during the evening and nighttime [17]. Therefore, the secondary objective is to explore the correlations between variations in serum 25(OH)D and indicators of sleep timing, sleep quality and general levels of fatigue.

3. STUDY DESIGN

A pilot study with a between-subjects manipulation is proposed for the current study design. The type of light (artificial UVB supplementation vs. no artificial UVB supplementation) is manipulated between subjects. In both the intervention group and the control group, repeated measures are employed during a sampling period of 8 weeks. When possible, randomization over the two groups will be conducted (see section 5.2 for more information). Figure 1 gives a graphical representation of the study design.

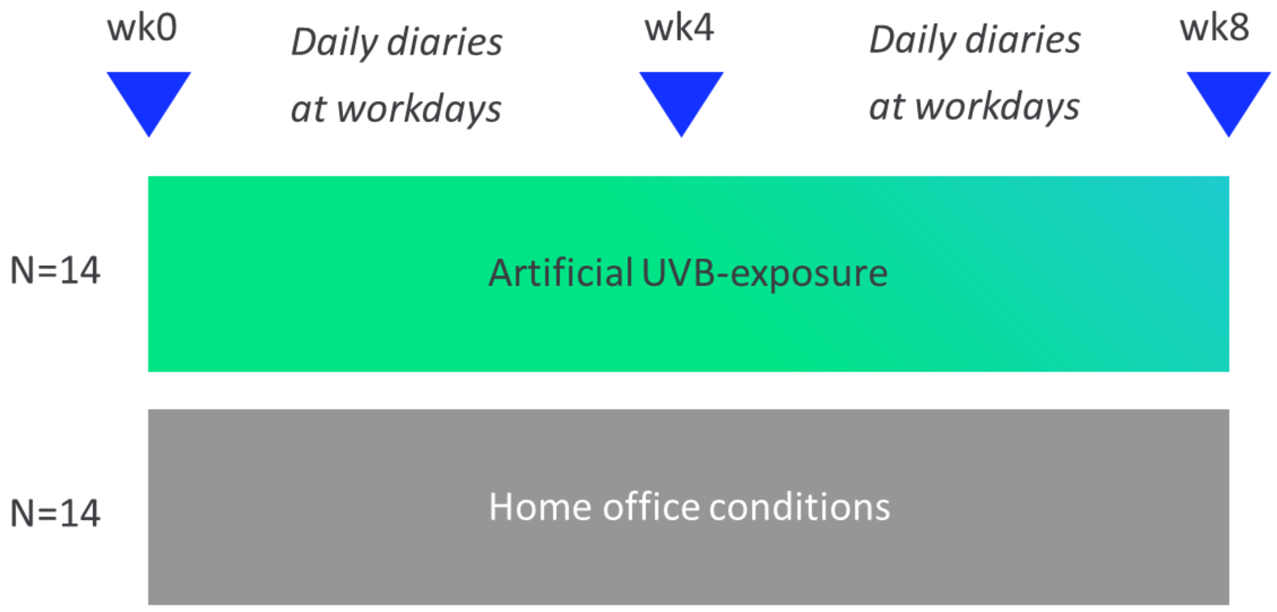

Figure 1. Graphical representation of the study design

For the primary objective, the serum 25(OH)D will be investigated. At the start of the study (day 0), participants will be asked to donate 4 ml blood to determine the baseline 25(OH)D levels in their blood. Subsequently, the light intervention is installed at the desks in the home office of participants in the intervention group delivering the ultra-low dose of UVB during daytime. The control group will not be exposed to artificial UVB radiation and will not receive a lamp at their desk.

The blood sampling and serum 25(OH)D analyses will be repeated in Week 4 and Week 8 on pre-scheduled morning time slots, preferably the same day for all participants, and is conducted by Diagnostiek Voor U (DVU) as a formally recognized health-care provider.

The self-reports on food intake (only vitamin D related), subjective sleep timing and quality, and general levels of fatigue will be investigated through (validated) questionnaires. Both the control and intervention group will receive the questionnaires electronically (via LimeSurvey) in the mornings during which blood sampling is also conducted. The second and third questionnaire contains an option for the participant

to give any comments or feedback about the study (procedure and intervention) to gain some additional insight in their study experience. Table 1 gives an overview of the frequency and timing of the data assessments. These assessments are all conducted in both groups.

Table 1 Overview of the frequency and timing of the different questionnaires and serum 25(OH)D level measurements

	Day 0	Day 29-31	Day 58-60
General information	X		
Serum 25(OH)D	X	X	X
Sleep timing and quality (based on [44 and [45])	X	X	X
General fatigue → Checklist Individual Strength [43]	X	X	X
Food intake (vitamin D related), based on [46]			
Feedback/comments (optional)		X	X

3.1 Duration of the study

The study protocol consists of a baseline measurement (Day 0) and an 8-week intervention period. Three data assessments will be conducted in day 1 of week 0, day 29-31 of week 4 and day 58-60 of week 8. Each data assessment is expected to take about 20-30 minutes. In addition to these three measurement points, participants are asked to complete a short (<1 minute) diary each day about desk presence, time spent outside, and clothing. The study is scheduled for autumn or winter 2020. This season has been chosen as both serum 25(OH)D and outdoor UV intensity start to decline rapidly to negligible values in this period [39,40].

3.2 Setting of the study

This study will be performed at the home office setting because of the high frequency of working from home during the COVID19 pandemic. The intervention group will plan a meeting with the lamp technician at the start of the study who will install the lamp in their home office and give explanation about how the lamp works. Blood sampling will be conducted at the DVU location in Eindhoven and questionnaires will be completed at home (online via the secured environment of LimeSurvey).

4. STUDY POPULATION

4.1 Population base

The population base exists in total of 1612 healthy people living in/around Eindhoven (18+).They will be selected according to the in- and exclusion criteria below (see section 4.2 and 4.3).for the possibility to participate in the study by an independent participant recruitment agency (CG selections). After approval of the criteria via e-mail, eligible participants will be contacted by CG selections via telephone to confirm the in- and exclusion criteria again.

4.2 Inclusion criteria

The inclusion criteria include:

- Age over 18 years old
- Fitzpatrick skin type II or III
- Living in/around Eindhoven
- Desk presence (at the home office) of at least 2,5 days per week during the 8 weeks study
- Medically fit to work the hours as contractually agreed
- Finding it no problem to have blood drawn (at Diagnostiek Voor U) three times over a period of 8 weeks

4.3 Exclusion criteria

The exclusion criteria include:

- Current pregnancy, breast feeding or a desire to become pregnant
- Having children at home aged 10 years or younger
- Having malignant skin conditions in the past or currently
- Photosensitive medical conditions or photo-sensitising drugs
- Users of medicines and/or cremes mentioning in the prescription as side effect extra sensitivity to the sun / interaction with sun exposure
- Planned use of sun beds, or sunbed use during the past 4 weeks
- Currently taking or planning to take oral vitamin D3 supplements or have been taking D3 supplements during the past 4 weeks.
- High vitamin D levels at the start of the study (>375 nmol/L) which need medical attention [41]

4.4 Sample size calculation

In order to estimate whether an ultra-low daily dose of artificial UVB (~0.5 SED) complying to the photobiological safety requirements for general lighting could be effective in raising serum 25(OH)D,

simulations based upon Diffey's model were conducted [42]. The simulation results indicate that when the foreseen intervention dose (0.45 SED) is applied in the optimal conditions (evenly over 8 hours and 20 minutes and 10.5 % body surface area exposure), a maintenance of summer serum vitamin D3 levels could be realized. Figure 2 represents the simulation (Exposure: 0.45 SED; Body Surface Area (BSA): 10.5%, front face (3.5%), 2 x back lower arms (3%), 2 x backside hand (3%), neck (1%)) as well as the effect of such an intervention in the foreseen study timespan of eight weeks. For the study it is expected that at optimized conditions a difference of about 17.6 nmol/L between intervention and control group can be realized in those 8 weeks. Having said that, the expected variance in actual exposure each day will be larger based upon the analysis of variations in exposure duration (desk occupancy) and body surface area exposure. The estimated impact of exposure to a minimum 0.225 SED and a body surface area % minimum of 6.5%) on serum vitamin D3 levels are depicted in Table 2.

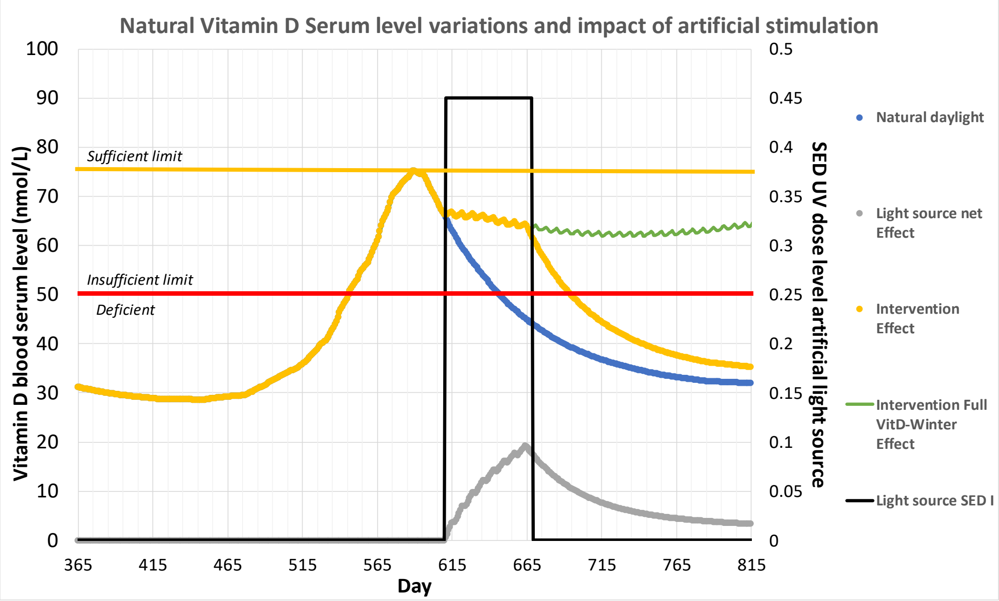

Figure 2: Simulations results conducted to estimate the impact of a 8 week UVB exposure of office workers with input parameters (10.5% skin exposure: front face (3.5%), 2 x back lower arms (3%), 2 x backside hand (3%), neck (1%)). At start: 66.4 nmol/L; End intervention group: 61.7 nmol/L (- 4.7 nmol/L); End Control Group: 44.1 nmol/L (-22.3 nmol/L); Expected intervention effectiveness: +17.6 nmol/L. The UVB dose is evenly (UVI=0.06) given over a period of 8 hours and 20 minutes during the workday. This figure is based on exposure during 5 working days.

Table 2: Simulation results for expected serum vitamin D3 levels at reduced body surface area (BSA%) exposure and intervention dose (0.225 SED (50%)).

		**Estimated Serum VitD Intervention Effect (nmol/L) compared to control group**		
**Variables**		**Extrapolated Intervention Effect**		**Extrapolated End-Effect (April)**
Daily Exposure (SED)	*Body Surface Area Exposure (%)*	*After 4 weeks*	***After 8 weeks***	*@ minimum of serum VitD control group*
0.45	10.5	+12.4 (+23%)	**+17.6 (+40%)**	+32 (+100%)
0.45	6.5	+8.8 (+17%)	**+13.6 (+31%)**	+20 (+60%)
0.225	10.5	+6.2 (+12%)	**+8.8 (+20%)**	+16 (+50%)
0.225	6.5	+3.8 (+7%)	**+5.5 (+12%)**	+10 (+30%)

These modelling results suggest that ultra-low doses of continuous UVB exposure for eight weeks of use are able to maintain serum 25(OH)D levels in healthy people compared to people who will not receive this exposure (control group). Moreover, the modelling results indicate that the expected differences are in line with effects found in earlier studies [33-38].

A sample size calculation using Gpower was performed assuming that the intervention will reach a (conservative) difference of 8.8 nmol/L between the intervention and the control group (situation 3 in Table 2) at the final measurement point (at 8 weeks). This would mean that the decrease in the control group from baseline to week 8 would be -22.3 while in the intervention group this would be -13.5 (difference of 8.8 nmol/L). The sample size calculation is based on the statistical analysis conducted for the primary objective, namely the difference in serum 25(OH)D between the intervention and control group at the final measurement point, taking into account an alpha of 0.05 and a power of 0.90. The standard deviation within one group is estimated based on a previous similar study which also implemented low-dose UVB exposure to increase serum 25(OH)D in a sample of 20 healthy participants, albeit over a week period using three full-body UVB sunbed sessions [35]. In the post-measurement of this study, they found a significant increase in serum 25(OH)D of 13.9 nmol/L. One month after the end of the UVB intervention they still found a significant increase of 8.8 nmol/L, which is a similar difference we expect in the intervention group compared to the control group. The standard deviation for the power calculation was therefore based on the standard deviation on the difference score found in the sample in this study (similar sample size and similar changes in serum 25(OH)D), namely 8.4 nmol/L. The sample size for detecting a difference of 8.8 nmol/L assuming a standard deviation of 8.4 nmol/L in each group would be 17 participants in each group (see figure 3 below). As we currently only have the availability of 14 lamps, we will choose for 14 participants in each group, and aim to find a larger effect, which is not unlikely when on average, there is somewhat more than 50% desk occupancy and/or if we reach a higher exposure surface area.

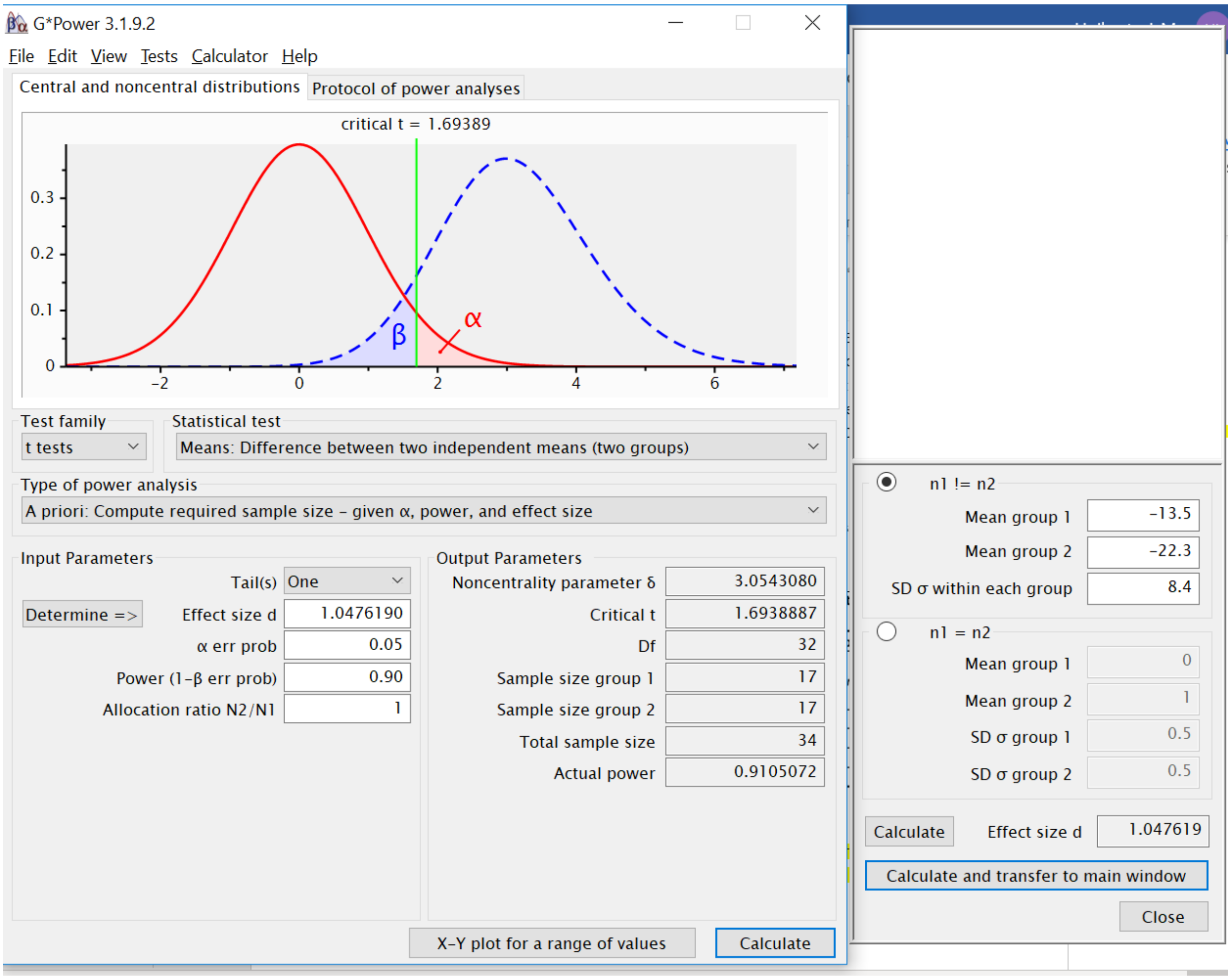

Figure 3. Sample size calculation for change in serum 25(OH)D at 8 weeks

5. TREATMENT OF SUBJECTS

All home office workers who will participate in the study will be asked to continue their work as they are used to do. During the workday all participants are exposed to the illumination of the existing general indoor lighting in their home. Depending on their desk location, participants may be exposed to daylight entering the office space through windows. This additional light will not contain UVB irradiance as UVB irradiance is absorbed by the window glass.

In addition to the standard lighting the **intervention group** will receive an additional personal desk-based luminaire that is positioned at the side of participant's home desk. The participants in the intervention group will be asked to be seated closer to one side of the desk, where the free floor standing luminaire is positioned, in order to sit more effectively under the luminaire. The area to be seated will be marked on the desk with stickers and tape. Figure 4 demonstrates the desk-based intervention lighting device placed at the side of the desk. Care is taken that the distance between the participant receiving a luminaire and the neighbor (non-participating) colleague/family member is more than 1.6 m to prevent that the (non-participating) colleague/family member is exposed to the ultra-low doses of UVB. The designated workspace of the control group is not altered for the purpose of the study. Control participants will experience only the existing general lighting in their home office).

The desk-based luminaire will emit a continuous ultra-low and safe daily artificial UVB irradiance (actinic UV) of less than 6 mW/m2 at 20 cm resulting in a received dose by the office worker sitting at his/her desk of about 0.45 SED ((UVI = 0.06) during office hours. The lamps are turned on automatically at 8.50 and off at 17.10 hr. A longer (over)-exposure is therefore not possible. Both groups can do their daily work as they are used to do. Both the participants and the people living in their home will be advised to respect the indicated minimum application distance (80 cm). This minimum application distance keeps the Actinic UV exposure within Risk Group Exempt limits, which allows for a continuous exposure of 30000 sec per day (8 hrs. and 20 minutes) according to the IEC62471 (see form *D6_2 Photobiological safety IEC 62471 ed 1.0*).

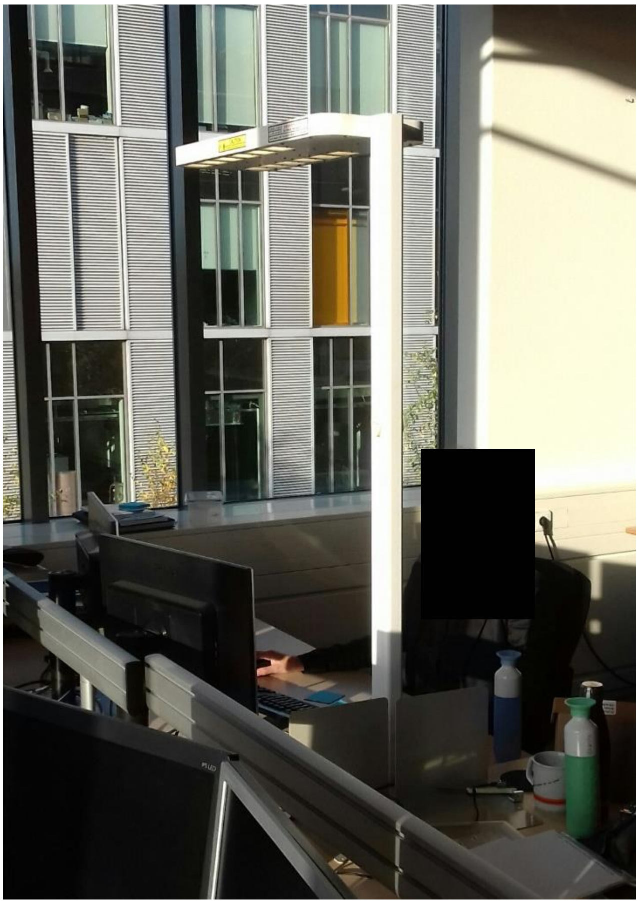

Figure 4. A picture of a currently designated workspace equipped with the desk-based lighting device.

6. INVESTIGATIONAL PRODUCT

6.1 Technical description

In order to investigate the effectivity of adding **ultra-low** dose(<0.5 SED, UVI =~0.06), but safe (according to the IEC62471) UVB irradiance in a general office lighting solution, a special lighting research device is developed according to the general lighting guidelines with respect to photobiological safety, mechanical and electrical requirements. The newly developed luminaire is based on a released, commercially available Free Floor Standing product (https://www.lighting.philips.com/main/prof/indoor-luminaires/free-standing/smartbalance-free-floor-standing and D6_5 CE declaration of commercial available smart_balance). The standard product is modified in such a way that

- Uplight is removed
- Downlight is reduced
- UVB light is added
- Light exit window is replaced by a UVB resistant version
- Drivers are replaced in line with new led configuration
- Bluetooth control is added to set the on-off rhythm and level.
- The level can only be configured by trained people and within safe limits.

The special lighting device is designed for research purposes towards new opportunities for general lighting and will not be commercialized as is.

The newly-developed luminaire-prototype emits visible and a very low amount of invisible (UVB) irradiation exposing unprotected skin and eye, and enabling natural processes that occur when the body is exposed to daylight; Therefore special attention has been dedicated to the photobiological safety as this is seen as the major modification of the free floor standing luminaire:

- Direct view to the naked UVB LED is prevented by the diffuser and in the event the diffuser is removed from the head, the UVB LEDs go off.
- Over exposure is prevented by limitation of the maximum UVB output by a firmware setting in the driver and a real time clock automatically turns on and off the light output (30.000 s).
- The personal desk-based lighting intervention will be checked on UVB irradiance levels on a regular basis to ensure a consistent dose during the pilot study. The relative stability will be monitored by a point measurement via solar meter model 6.5 (UV) at 20 cm of the head of the luminaire. Additionally, a continuous daily dose measurement is conducted by placing wear shade dosimeters at the desk.
- Device labeling and instruction to the user to not stare in the luminaire head and to prevent prolonged exposure to distances shorter than 80 cm.

Since the UV irradiance is the innovative part in this device, Prof A. Webb, Atmospheric Radiation from Manchester University has run an independent test (see *D6_1 Analysis of Signify Luminaires_FINAL*) to confirm the radiance measurements made by the lighting quality center in Eindhoven, to ensure safety to the users. The main conclusion from this report is that “the luminaires meet all safety requirements for intended use if the affixed safety label states a safe distance of 800 mm. People who do not wish to receive radiation from the luminaires should be seated > 1.6 m from the point beneath the center of the LED housing, as this is the distance at which the irradiance is reduced to 10% of the central maximum and can be considered negligible from the perspective of biological effects.”

For more information and details on the desk-based lighting device we refer to the investigator’s brochure and associated documents (D1 *investigator’s brochure and risk analysis_v2, D6_2 Photobiological safety IEC 62471 ed 1.; D6_3 Example of biological safety evaluation report HJ11814*;).

The personal desk-based lighting device carries a CE mark with document no FS484F UV-B DoC (*D6_8 CE Declaration of Conformity (FS484F UVB DoC*)

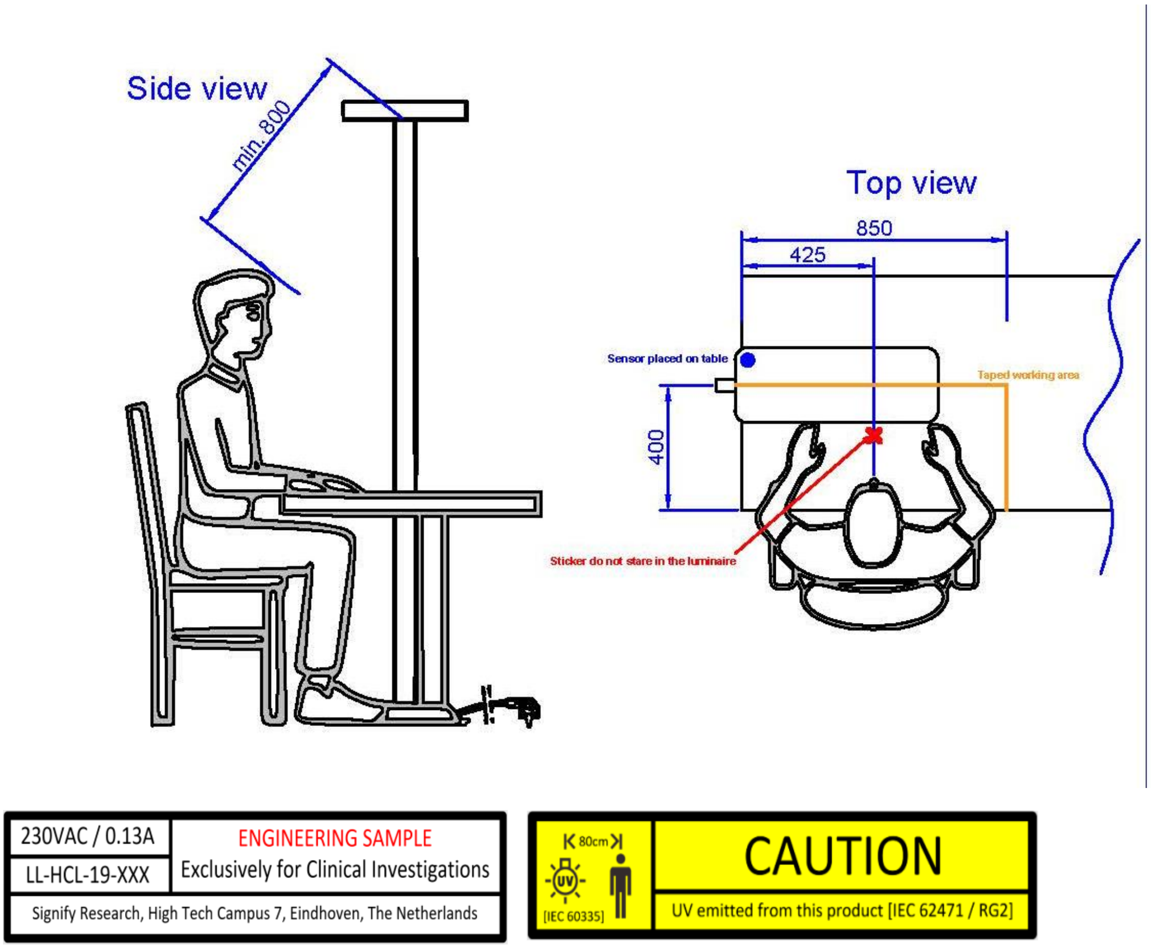

Figure 5. Illustrated (safe) use of the luminaire and the caution labels

6.2 Operating behavior

First day luminaire behavior

After the first blood sampling has been taken place, the luminaire will be powered by putting the plug in the mains socket by the technical team. When the luminaire is connected to power it will start with dimmed white light and no UVB. A small amount of white light is emitted to indicate that the luminaire is ON. At noon or when powered after 13.00 hr, the next morning, the luminaire will switch ON (full brightness and UVB) according the preset schedule: ON at 8.50 hr and OFF at 17.10 hr (both the white and UV-B light will turn off). This behavior will also occur in case of a power cycle / disturbance in power.

Luminaire behavior during the study

The luminaire will automatically switch ON (full brightness and UVB) at 8.50 hr according to the preset schedule. At 17.10 hr both the white and UV-B light will turn off. This will be repeated for all days during the study period as long as the luminaire is powered. The simultaneous switching on of the visual white light and the UVB lights is an additional visual cue to the participant and other people living in the home to remind them that there is UVB irradiance and that directly looking into the luminaire should be avoided.

The luminaire is equipped with caution labels and a user manual will be supplied to the participant to facilitate proper use of the luminaire and easy access to a contact person if questions pop up.

7. METHODS

7.1 Study parameters/endpoints

7.1.1 Main study parameter/endpoint

For the primary objective, the serum 25(OH)D will be investigated. The blood sampling (one tube of 4 ml blood per measurement) will be conducted by a medically trained person of DVU as a formally recognized health provider. The actual serum 25(OH)D analyses will also be conducted by DVU. This measure will be collected at baseline, after 4 weeks of the intervention, and after 8 weeks of the intervention.

7.1.2 Secondary study parameters/endpoints (if applicable)

Secondary parameters include self-report measures:

- A validated survey on general fatigue will be filled in at each measurement point, the Checklist Individual Strength [43]. In addition, several items on sleep timing and quality, averaged over the past two weeks (as in the Checklist Individual Strength) will be completed in the survey. The questions on sleep timing and quality are based on the Consensus Sleep Diary and the Munich Chronotype Questionnaire (but asked as averaged over the past two (instead of four) weeks; see [44] and [45]). In addition, vitamin D related food intake during the past four weeks will be collected (based on [46]). The questions will be programmed in the LimeSurvey tool used at the Technical University of Eindhoven. Responses on these questions will only be saved locally on the encrypted TU/e server.
- Desk presence data, time spent outside, and exposed body surface area: These data come from self-assessment which will be facilitated on hard-copy pre-defined tables during the luminaire placement. In this diary, participants will report on the amount of time (hours and minutes) spent at the desk in the morning hours (8:50-13:00) and afternoon hours (13:00-17:10). In addition, they report the time spent outside (hours and minutes) in daylight between 8:50 and 17:10. They will also report this on Monday for the previous weekend days. Finally, they report on the body parts that are not covered by clothing (e.g. neck, face, under arms, upper arms, hands etc.). Participants will be asked to upload a scan/photo of the daily diaries every week on Friday in the same LimeSurvey tool as is used for the other questionnaires.

7.1.3 Other study parameters (if applicable)

- Age, gender, and amount of working hours each day will be assessed at the start of the baseline week in order to enable description of both groups. Questionnaires will

also be programmed in LimeSurvey and sent via a link to participants. Subsequently, participants can fill in the questionnaires on their own (work) laptop.

7.2 Randomisation, blinding and treatment allocation

For a full visual overview of the allocation procedure see Figure 6. The independent agency CG selections will make a selection of eligible participants based in the in- and exclusion criteria (see sections 4.2 and 4.3). A list with eligible participants who are interested in participating in the study is mailed to the responsible researcher at the TU/e who will perform block randomization for assignment to the conditions. However, in case insufficient participants are available who work at least 2.5 days at their home office, selection for the intervention group will then be based on presence at the desk (at least 50%). This selection is believed not to influence the primary outcome parameter (25(OH)D serum levels) and as such the conclusions around the primary objective: Participants will not receive any (noteworthy, see [39]) UVB exposure (that could influence the serum 25(OH)D) unless they are allocated to the intervention group. In the statistical analysis, care will be taken to control for potential group differences in desk presence, time spent outside and vitamin D intake by food.

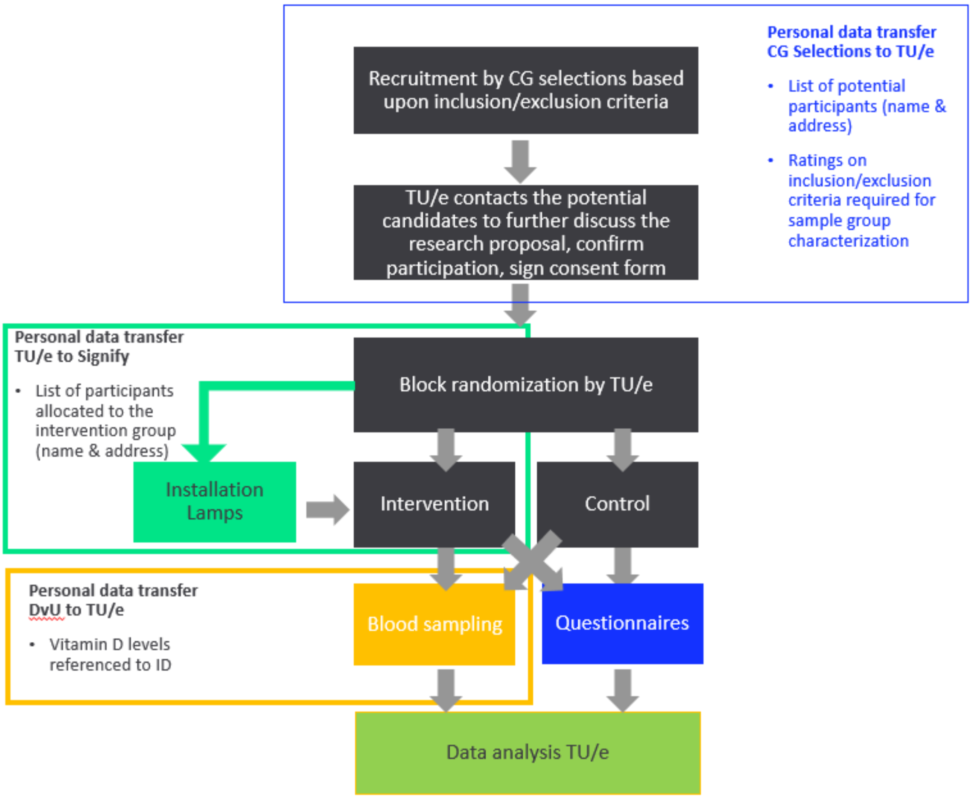

Figure 6. Allocation procedure

In addition to randomization, blinding is frequently applied to prevent that participants know they are in the control or intervention group. For this specific pilot, only 14 luminaires are available. These luminaires will be placed at the side of the participant's desk when allocated to the experimental group. As such the participant is aware that he or she will receive UVB. Blinding by reducing the sample size to 14 instead of 28 has been considered. However, to prevent that the data can be allocated (identified) to individual persons and to have sufficient power in the study, the total number of participants is estimated to be >25 in total. Since the 25(OH)D levels will not be affected by a pseudo-randomization and non-blinding strategy, it has been chosen to secure privacy of the participants and prioritize a sufficient sample size, and blinding is compromised.

Secondary objective

The secondary objectives focusing on sleep and general feelings of fatigue on the other hand can be influenced by prior knowledge to allocation to the intervention and control. Therefore, we will include these for exploratory purposes and we will only explore whether variation in vitamin D status in both groups correlates with variations in sleep and general feelings of fatigue, instead of investigating the direct effect of the intervention in these indicators.

7.3 Data treatment

7.3.1 General

CG selections will send a list of names, e-mails, and addresses to the researcher at TU/e who will further inform participants (Dr. Laura Huiberts). This data is shared by CG selections via their secured customer portal. The TU/e researcher responsible for informing the participants about the study and randomizing the participants (Dr. Laura Huiberts) will construct an account with secured password in this portal where this data can be accessed.

After randomization, names, addresses and phone numbers of participants in the intervention group will be send from TU/e to Signify only with the goal to install the luminaire at the home of participants (in the intervention group). Participants will also receive a participant ID at the beginning of the study by the responsible researcher at the TU/e (Dr. Laura Huiberts). They will use this ID to complete all online questionnaires so that no names or e-mails are logged by the questionnaire tool. DVU (responsible for the blood sampling) will also receive these participant ID's so that they can transfer the 25(OH)D serum levels to TU/e anonymously (only using the participant ID's). An encrypted file saved at the secured laptop of the TU/e researcher will contain the key regarding assigned ID numbers and participant names. This file is only accessible for the TU/e research team (Dr. Laura Huiberts, Prof Yvonne de Kort, and Dr. Karin Smolders). Data from the questionnaire tool (see section 5.3.4.) and the blood samples (see section 5.3.3) will be double coded (pseudonymized) by an independent researcher

of the HTI department and will send the double coded data (without initial ID numbers) back to the investigator for the analyses (Dr. Laura Huiberts). The pseudonymized full data set will be stored for eat least 15 years at the encrypted TU/e server. An encrypted file with the key/link between the assigned number/code and participant ID's will be saved and stored in a secured environment at the HTI department and is only accessible to the appointed researcher and can be accessed on request by the responsible TU/e research team if needed. There will be no reporting on individual results, only on the group level (aggregated data). Pseudonymized data will only be accessible to the investigators from the TU/e (see above) and will not be shared with Signify (or any other party/researcher apart from the assigned TU/e researchers: Prof. Yvonne de Kort, Dr. Karin Smolders, and Dr. Laura Huiberts). Reports on the aggregated results will be shared with Signify (e.g. means, SD's, min-max values, graphs/bar charts of these values, and aggregated statistical tests to test the hypotheses).

7.3.2 Data treatment of inclusion/exclusion criteria

For the inclusion/exclusion screening, CG selections will send an email to potential participants in their participant base (see section 4.1) They will be asked to confirm whether they can be included or not based on the in- and exclusion criteria mentioned in the e-mail, without mentioning the specific reason. This prevents processing needless personal details. In case participants can and want to participate, they will sign an informed consent form provided by CG selections approving for transfer of their name, e-mail, and phone number to the responsible researcher from TU/e.

7.3.3 Main study parameter: Vitamin D3 serum levels

The blood sampling procedure and serum analysis will be conducted by Diagnostiek Voor U Eindhoven (DVU) as a formally recognized health provider. DVU will send the blood sample results combined with the initially assigned Participant ID's via an encrypted e-mail to the HTI researcher who will double code (pseudonymize) this data before sending it to the data-analyst (Dr. Laura Huiberts). Participants give consent for DVU sharing this data with the TU/e researcher via the consent form. No diagnosis or advice can and will be given based on the serum vitamin D3 levels. If participants have any questions regarding the gathered blood values, they can turn to their general practitioner or contact DVU. Serum 25(OH)D values will be stored for long-term at the local secured TU/e server (15 years). DVU U will destroy the actual blood samples 5 days after the blood analyses have been conducted at the laboratory. DVU will store the coded vitamin D blood values for a period of 20 years.

7.3.4 Secondary parameters on sleep and general fatigue

Participants will receive a participant ID from the researcher at the TU/e which they (the participant) will use to complete the questionnaires. The online tool Limesurvey will be used to complete the questionnaires. The questionnaires on sleep and fatigue will be completed via this tool three times (baseline, measurement 1, and measurement 2). The daily presence diary (completed daily on paper) will be uploaded (scan or photo) via Limesurvey as well every week using the same participant ID. Limesurvey is a locally installed and secured tool currently used at the TU/e for conducting online questionnaire research. Data collected via this tool will only be saved locally at the secured TU/e server. Participants will only fill in their participant number and the answers to the questionnaires. No other personal data that can be tracked back to a person (e.g. names, e-mail addresses, residence, IP numbers etc.) will be probed in these questionnaires. Limesurvey data will be downloaded at the end of the study by the independent HTI researcher who will pseudonymize the data before it is sent (together with the serum 25(OH)D) values) to the researcher who will conduct the analyses (Dr. Laura Huiberts).

7.4 Study procedures

The selection and recruitment will take place by a stepwise approach:

1. CG selections will send out an e-mail to the population base in Eindhoven and surroundings (18+) including the sum-up of the in- and exclusion criteria. Via an e-mail to CG selections, participants can express interest in participation in case they meet the in- and exclusion criteria. In the email from CG selections, participants also give consent to send personal information (name, phone number, home address) to TU/e.
2. CG selections will call participants who are interested in participating and double-check the criteria for participation via phone. Participants will give oral and written permission to CG selections to share their e-mail and phone number with the TU/e researcher (Dr. Laura Huiberts).
3. If the employee has given permission to share the e-mail address with the TU/e, the TU/e will approach the employees and inform them on the procedure of the pilot study towards the effects of artificial UVB on vitamin D levels by e-mail. The participant will sign the consent form after receiving the oral instructions about the study and reading the participant information.
4. Eligible participants will be randomized to the intervention or the control group.

Before the start of the execution of the study protocol, participants who adhere to the inclusion criteria will be contacted and informed about the study by the research team and provided the opportunity to ask questions. At the end of this meeting, participants are asked to read the participant information again in their own time and send back the signed informed consent form if they decide to participate. In this consent form, participants also sign for transfer or their home address (contact details) to Signify, only with the goal to make an appointment to install the luminaire at their home office. Participants can ask any questions regarding the study via e-mail or phone before deciding to participate. Furthermore, the researcher will emphasize that it is always possible to stop at any time during the study without the necessity to explain choices, without any further consequences. After deciding (within 3-5 days after being informed by the researcher) to participate and signing the consent form, the researcher will inform the participants on which three days the data collection will occur (blood sampling and main questionnaire). Ideally, the blood sampling occurs on the same day of the week, at approximately the same time. Participants will inform the researcher at which timeslot they are available for the blood sampling, and if desired, the researcher can plan an appointment at DVU for the participant. Alternatively, participants can also make an appointment at DVU themselves (online) where they choose a time that fits in their schedule. In case enough participants meet the in- and exclusion criteria (at least 28), block randomization will be used by the responsible researcher to assign participants to the two conditions. In case there are insufficient participants who can guarantee at least 2.5 days of desk presence in the home office, participants with at least 2.5 days of desk presence will first be assigned to the intervention condition (based on timing of sending back the informed consent form) and remaining participants will be assigned to the control condition.

Participants will receive the first questionnaire via an e-mail link in the morning of the first assessment day (first blood sampling). The outcome of the first blood sample will be awaited before starting the intervention (installing the luminaire at the desk). This is done so that participants with high vitamin D levels that need medical attention (>375 nmol/L, [41]) can be excluded from the study before receiving any extra UVB exposure (further increasing their vitamin D levels). Although we do not expect such high vitamin D levels, if it occurs, participants will be contacted by the responsible researcher explaining that participating is not possible due to high vitamin D levels in the first blood sampling test. Participants will also be advised to check their level again at their general practitioner to see if a medical reference is needed. In this case, other eligible participants willing to participate will be contacted to ask if they still want to participate. These can replace the participant(s) who drop out.

Installing the luminaire will be conducted by a technical employee from Signify in the homes of the 14 participants in the intervention group. These participants will receive a separate consent form from Signify in which they give written consent that they received the luminaire and that they will not try to sell or open the luminaire, or transfer any information about the luminaire to others during the study period. During the installing process, participants will receive a short explanation about the luminaire (e.g. on and off times, how to switch it off if needed) and they will receive a short manual about the luminaire.

After the installation of the luminaires, the study starts where participants in the intervention group will be exposed to the luminaire between 8:50 and 17:10 during working hours on workdays. In the weekends and between 17:11 and 8:49, the luminaire will be off. This is an automated process. Participants in de control group will not receive the intervention luminaire and only complete the questionnaires and the blood sampling.

7.4.1 *The data assessment weeks*

Week 0, Week 4 and Week 8 are the data assessment weeks, employing the blood sampling and self-report measures (questionnaire via Limesurvey). The participants are also asked to fill in the logbook on a daily basis of which the data is uploaded on Limesurvey weekly (by scan or photo). Participants can also choose to complete the daily diary online right away daily.

7.4.1.1 Blood sampling to measure 25OHD serum levels

Blood will be sampled three times: in week 0, at pre-scheduled day in week 4, and at pre-scheduled day in week 8 at DVU. The laboratory will analyze the concentration of 25(OH)D in the blood using an immunoassay method. The accuracy of the used analysis method is 95%. Five days after the analysis the remaining blood samples will be destroyed as medical waste. DVU sends anonymous vitamin D values (using a participant ID) to the TU/e researcher who will pseudonymize the data after sending it to the data-analyst.

7.4.1.2. Questionnaires

Questionnaires to be found in F1 Questionnaires v2.0 20200515 will be programmed in Limesurvey (a locally installed tool used at the TU/e where only local storage of the data is enabled). The questionnaires are sent via a link to the participants' e-mail on the day of the blood sampling (in the morning). Subsequently, participants can fill in the questionnaires on their own PC/laptop. General levels of fatigue will be measured through the 20 items of the Checklist Individual Strength [43], sleep quality and timing are assessed through 19 items based on the Consensus sleep diary [44] and the Munich Chronotype Questionnaire [45]. The first online questionnaire (sent at day 1 in week 0) also includes questions related to the target group description like age, gender, and vitamin D related food intake (see section 5.1.3; food intake will also be measured in week 4 and 8). Participants will receive a reminder to complete the questionnaire towards the end of the working day if they have not completed the questionnaire yet. They can also do this at home since no IP addresses are saved by the Limesurvey tool.

7.4.1.3 UV-dose measurements

Because the major determinant of artificial UV exposure will be time spent at desk each week and body surface exposed area, this will be recorded daily using a daily dairy (see Figure 7), in which all subjects (also those in the control group) will self-report desk presence time and time spent outside in the morning and afternoon of their working day, and give an indication how much body surface areas was exposed during that day. Time spent outside during daylight in the weekend will also be reported on Monday.

Together with KNMI data we will be able to estimate the natural UVB exposure of the participant and provide for an analysis whether both groups behave similarly in their natural UVB dose. However, natural UVB exposure is expected to be minimal during the study months in autumn and winter (see [39])

Participants will gently be reminded to fill in the daily diary every day towards the end of the day. At the end of the week, they will receive a link to upload a scan or photo of the completed daily diaries or they can do this daily via a link provided at the start of the study.

Daily diary week 1										
**Estimated Desk Presence & Estimated Time Outside**										
Time/Day	**Monday**		**Tuesday**		**Wednesday**		**Thursday**		**Friday**	
	**Desk presence**	**Time outside**	**Desk presence**	**Time outside**	**Desk presence**	**Time outside**	**Desk presence**	**Time outside**	**Desk presence**	**Time outside**

08:50-13:00	__ hour(s) __ min.	__ hour(s) __ min.	__ hour(s) __ min.	__ hour(s) __ min.	__ hour(s) __ min.	__ hour(s) __ min.	__ hour(s) __ min.	__ hour(s) __ min.	__ hour(s) __ min.	__ hour(s) __ min.
13:00-17:10	__ hour(s) __ min.	__ hour(s) __ min.	__ hour(s) __ min.	__ hour(s) __ min.	__ hour(s) __ min.	__ hour(s) __ min.	__ hour(s) __ min.	__ hour(s) __ min.	__ hour(s) __ min.	__ hour(s) __ min.

**Weekend exposure**	
Total time outside between 8:50 and 17:10 **Saturday**	___hour(s) ___ min.
Total time outside between 8:50 and 17:10 **Sunday**	___hour(s) ___ min.

**Body Surface Area (BSA) exposure (Mark with X which body area is exposed)**					
	**Monday**	**Tuesday**	**Wednesday**	**Thursday**	**Friday**
Front Face					
Neck					
Hands					
Lower arms					
Upper arms					
Other, namely					
**To be filled in by study manager**					
% occupation					
%BSA					
% time natural UV-B					

Figure 7: Example of the diary to be filled in daily

7.5 Withdrawal of individual subjects

Subjects can leave the study at any time without giving a reason if they wish to do so without any negative consequences. In the event that a subject stops before data assessment week 4, an attempt will be made to recruit a new volunteer (if available) who can start his/her first measurements in week 4.

7.6 Premature termination of the study

Premature termination of the study is not expected to happen as no serious adverse effects are expected due to the lighting intervention or study protocol. Nevertheless, in case of a serious adverse effect has taken place, steps will be implemented to reduce these adverse effects. If necessary, the study will be terminated temporarily, until improvements have been implemented. If significant adverse effects persist, the study may be terminated prematurely. In that case, all participants and the reviewing accredited METC, will be informed.

8. SAFETY REPORTING

8.1 Temporary halt for reasons of subject safety

In accordance to section 10, subsection 4, of the WMO, the study will be suspended if there is sufficient ground that continuation of the study will jeopardise subject health or safety. The accredited METC will be notified without undue delay of a temporary halt including the reason for such an action. The study will be suspended pending a further positive decision by the accredited METC. The responsible investigator will take care that all subjects are kept informed.

8.2 AEs, SAEs and SUSARs

8.2.1 Adverse events (AEs)

Adverse events are defined as any undesirable experience occurring to a subject during the study, whether or not considered to be related to the investigational product, trial procedure or the experimental intervention. All adverse events reported spontaneously by the subject or observed by the investigator or his staff will be recorded and reported to the sponsor.
We don’t expect any adverse events due to the lighting intervention as it is thoroughly tested by Signify test centres and an independent party. Although very unlikely, exposure to the desk-based lighting device might result in discomfort (e.g. visual discomfort or eyestrain, headaches/migraines, or physical complaints due to working at the side of the desk). Blood sampling might lead to distressing effects or increased work pressure might arise as a result of the time investment needed on providing data for the research. All AE’s will be registered by the responsible investigator. Participants can contact the investigator or leave any comments/feedback/complaints in all of the questionnaires in the study

(weekly). In case any AE's occur that need action, the responsible researcher will contact the participant (if he/she did not do that him/herself) and discuss whether the study should be discontinued. Reference to the participant's general practitioner is advised in case of physical complaints that need medical evaluation. At the start of the study, participants will receive a letter for their general practitioner with information regarding the study and a contact telephone number and e-mail at which they can reach the responsible researcher for more information. Participants can take this letter to their general practitioner if needed.

8.2.2 Serious adverse events (SAEs)

We do not expect SAEs from this study. In case they will occur, they will be reported as described below.

A serious adverse event is any untoward medical occurrence or effect that

- results in death;
- is life threatening (at the time of the event);
- requires hospitalisation or prolongation of existing inpatients' hospitalisation;
- results in persistent or significant disability or incapacity;
- is a congenital anomaly or birth defect; or
- any other important medical event that did not result in any of the outcomes listed above due to medical or surgical intervention but could have been based upon appropriate judgement by the investigator.

An elective hospital admission will not be considered as a serious adverse event.

The responsible investigator will report all SAEs to the sponsor without undue delay after obtaining knowledge of the events.

The sponsor will report the SAEs through the web portal *ToetsingOnline* to the accredited METC that approved the protocol, within 7 days of first knowledge for SAEs that result in death or are life threatening followed by a period of maximum of 8 days to complete the initial preliminary report. All other SAEs will be reported within a period of maximum 15 days after the sponsor has first knowledge of the serious adverse events.

8.2.3 Suspected unexpected serious adverse reactions (SUSARs)

Not applicable.

8.3 Annual safety report

Not applicable.

8.4 Follow-up of adverse events

All AEs will be followed until they have abated, or until a stable situation has been reached. Depending on the event, follow up may require additional tests or medical procedures as indicated, and/or referral to the general physician or a medical specialist. SAEs need to be reported till end of study within the Netherlands, as defined in the protocol (section 6.2.2.).

8.5 Data Safety Monitoring Board (DSMB) / Safety Committee

The WMO requires on-site monitoring for drug related research conform the ICH-GCP regulation (2005/28 EG). However, in case the clinical trial is not related to drugs and still WMO obligatory, monitoring is not legally required. The intervention of the proposed clinical trial is a lighting intervention and not a drug. Therefore, a request for exemption will be made.

9. STATISTICAL ANALYSIS

The data analyses consist of multiple steps. All data will be presented quantitatively.
As a first step, we will inspect the distribution of the variables of interest. A Shapiro-Wilk test will be conducted to inspect normality of the variables. If needed and possible, a transformation of the scores will be performed to adhere to the assumption related to normality for the statistical analyses. Subsequently, an outlier detection will be performed with outliers determined as values more than 3 SD's from the mean.
Next, we will provide the descriptive statistics for the experimental and control condition for the variables measured only at baseline (see Table 1), as well as for the dependent variables (i.e., the variables for the primary (serum 25(OH)D) and exploratory research objectives (fatigue and sleep), see Section 2.) for each of the measurement points. Comparisons of the variables at baseline will be investigated via independent samples T-test to examine whether there were significant differences between the two groups for any of the variables at measured baseline. If the data appears to be not normally distributed and no transformation is applicable, we will employ a non-parametric test (Mann-Whitney test).

Subsequently, the main research objective (i.e., effects of the light intervention on serum 25(OH)D at week 8) will be investigated using an ANCOVA analyses with the intervention (yes/no) as predictor and change scores in serum 25(OH)D as outcome variable. This analysis will further be controlled for average desk presence and time spent outside. In case data is non-normally distributed a non-parametric test (Mann-Whitney test) will be conducted taking into account the limitation that this analysis cannot control for the mentioned confounders. Analyses will only be conducted with data from participants with both the baseline measurement and the final measurement point available. An alpha level of 0.05 will be used as criterion.

After that, an explorative Linear Mixed Model (LMM) analysis will be conducted to examine changes in serum 25(OH)D over time (i.. e, first and second measurement, using baseline scores as a control variable). LMM analyses will executed because this type of analysis takes into account dependency of the data due to the repeated measures as well as potential missing values at any of the measurement points. In case a significant Intervention * Measurement interaction is found ($p < 0.05$), post-hoc comparisons are conducted to investigated differences between the intervention and control group at each of the two measurement points. In addition, we will conduct an exploratory LMM analysis in which we investigate the correlation between UVB dose (calculated based on desk presence and clothing) and serum 25(OH)D over time to examine whether there are large differences in these values based on the UVB dose participants received. These explorative analyses will also be controlled for time spent outside during daytime.

Last, exploratory LMM analyses will be conducted to investigate relations between the variation in serum 25(OH)D and variations in sleep duration, sleep quality, and general feelings of fatigue.
This will be established by running a LMM model with serum 25(OH)D as well as measurement point as predictor in order to account for changes in sleep patterns or fatigue levels over time. For visual inspection purposes, this will also be investigated separately in both the intervention and the control

group. This will be analyzed in this way due to a lack of blinding in participants which may create bias when directly investigating the effect of the intervention on fatigue/sleep.

10. ETHICAL CONSIDERATIONS

10.1 Regulation statement

The study will be conducted in accordance with the principles of the Declaration of Helsinki (Amendment of the 59th WMA General Assembly, Seoul, Korea, October 2008) in accordance with the Medical Research Involving Human Subjects Act (WMO) and conform General Data Protection Regulation (GDPR) effective:25 May 2018.

10.2 Recruitment and consent

The selection and recruitment will take place by a stepwise approach executed by an external party (CG selections) (see also section 7.4):

1. CG selections will send out an e-mail to the population base in Eindhoven and surroundings (18+) including the sum-up of the in- and exclusion criteria. Via an e-mail to CG selections, participants can express interest in participation in case they meet the in- and exclusion criteria. In the email from CG selections, participants also give consent to send personal information (name, phone number, home address) to TU/e.
2. CG selections will call participants who are interested in participating and double-check the criteria for participation via phone. Participants will give oral and written permission to CG selections to share their e-mail and phone number with the TU/e researcher (Dr. Laura Huiberts).
3. If the employee has given permission to share the e-mail address with the TU/e, the TU/e will approach the employees and inform them on the procedure of the pilot study towards the effects of artificial UVB on vitamin D levels by e-mail. The participant will sign the consent form after receiving the oral instructions about the study and reading the participant information.
4. Eligible participants will be randomized to the intervention or the control group.

Before the start of the execution of the study protocol, participants who adhere to the inclusion criteria will be contacted and informed about the study by the TU/e researcher (Dr. Laura Huiberts) and provided the opportunity to ask questions. At the end of this meeting, participants are asked to read the participant information again in their own time and send back the signed informed consent form if they decide to participate. Participants can ask any questions regarding the study via e-mail before deciding to participate. Furthermore, the researcher will emphasize that it is always possible to stop at any time during the study without the necessity to explain choices, without any further consequences. In case sufficient participants meet the in- and exclusion criteria, block randomization will be used by the

responsible researcher to assign participants to conditions. Otherwise, participants who have at least 2.5 days of desk presence at home (and meet the other criteria) will be assigned to the intervention condition first (based on timing of sending back the informed consent form) and the other participants will be will be assigned to the control condition.

Participants will receive the first questionnaire via an e-mail link in the morning of the first assessment day (first blood sampling). The outcome of the first blood sample will be awaited before starting the intervention (switching on the luminaire at the desk). This is done so that participants with high vitamin D levels that need medical attention (>375 nmol/L, [41]) can be excluded from the study before receiving any extra UVB exposure (further increasing their vitamin D levels). Although we do not expect such high vitamin D levels, if it occurs, participants will be contacted by the responsible researcher explaining that participating is not possible due to high vitamin D levels in the first blood sampling test. Participants will also be advised to check their level again at their general practitioner to see if a medical reference is needed.

Due to the nature of the study procedure and involved parties the participant will be asked to sign three consent forms three informed consents:

1. Consent on the privacy regulations and conditions from CG selections: https://www.cgresearch.nl/voorwaarden-en-privacy/ and permission to send personal data (name, phone number, home address) to TU/e for study participation.
2. Informed consent form for study participation (including permission to send contact details to Signify with the purpose of installation of the luminaire, and permission for DVU to share the serum vitamin D values with the research team at TU/e).
3. Written consent from Signify for receiving the luminaire and not trying to sell it / disassemble the luminaire (consent given at the installation of the luminaire)

10.3 Objection by minors or incapacitated subjects (if applicable)

Not applicable.

10.4 Benefits and risks assessment, group relatedness

10.4.1 Benefits

Expected benefits (particularly in intervention group) is maintenance summer vitamin D3 levels and through that an enhanced sense-of-wellbeing.

10.4.2 Possible burden

The main burden for participation in the study will be time consumption and slight discomfort to perform the blood sampling and to fill in the online questionnaire. Expected time consumption is estimated to be around 100 minutes maximum in total during the full 8-week study period. The desk-based lighting intervention and most of the measurements will be performed at current workspaces with minor interference with normal activities. Table 3 provides an overview of the estimated time investment per measurement type. The duration in Table 3 is excluding the travel time to and from DVU (three times), which depends on the home address of the participant.

Table 3 Overview of estimated time investment

Frequency	Measurements	Time
3x	Online questionnaire	10 minutes
3 x	Blood sampling	10 minutes
40x	Daily diary	<1 minute

10.4.3 Potential risk

While potential risks related to the lighting intervention are expected to be minimal, exposure to the personal desk-based lighting intervention may result in undesirable experiences such as experienced discomfort (e.g., visual discomfort or eyestrain) or headache. Additionally, the use of the study protocol might result in distressing effects in relation to an oversensitivity to UVB, unknown at the start of the study. In that case if the participant notices a potential distressing effect, he/she can stop immediately.

Negative effects of UVB are associated to the formation of cyclobutylpyrimidine dimers in DNA contributing to the mechanism of erythema and sunburn formation, generally occurring during prolonged exposure at very sunny days (UV-I > 5). To place the UV dose applied in this study in context, the dose received throughout a full working day would be equivalent to 10 minutes in bright sunlight at lunchtime on a summer day in Northern Europe. It is clearly **below the daily dose for the EU Safety Directive 2006/25/EC, and well below the erythema (sunburn) threshold for all skin types**. Individuals who suffer from photosensitivity disorders, take photosensitising medication or have had treatment for skin cancer will be excluded from the trial. The main identified risk factors associated with these photobiological safety aspects:

- exceeding the 0.7 SED per day limit due to long office hours
- direct (prolonged) viewing of the UVB LED at short distances
- lack of awareness of UVB irradiance from the source

have been mitigated by design, protective measures and information for safe use.

The contribution to risk of developing skin cancer by exposure to ultra low doses of artificial UVB during a 2-month period is considered to be negligible:

- The ultra-low daily dose is five-fold lower than the guideline to prevent sunburn for skin type 1.
- The recommendation of the KWF for required healthy sun exposure is in line with what is intended in this study.
- The potential protective effects of vitamin D to attenuate inflammation due to sunburn

In summary, the application precautions are considered to make the luminaire safe for the intended application, as described in the photobiological safety photobiological hazard (European directive 2006/25/EC), and the device can be regarded safe to use under application conditions according to IEC 62471 .

10.5 Incentives (if applicable)

The maximum compensation for participants is 125 euros. For each missing blood sampling sessions, 30 euros will be subtracted from the maximum compensation (unless there were any valid reasons not to do the blood sampling, e.g. being sick/ urgent interferences). For every missing main questionnaire (completed three times; in week 0, 4 and 8), 5 euros will be subtracted from the compensation. In addition, 2,50 euros will be subtracted from the compensation for each incomplete weekly questionnaire upload (including 5 short daily questionnaires).

11. ADMINISTRATIVE ASPECTS, MONITORING AND PUBLICATION

11.1 Handling and storage of the data and documents

A data sharing agreement between CG selections and TU/e will be constructed regarding data sharing of participant names, phone numbers, e-mail addresses and home addresses. After a selection of participants is made based on the in- and exclusion criteria and interest to participate, participants will be contacted by the TU/e researcher for explanation of the study, participant information, and signing the consent form. All participants who signed the consent form and are allocated to one of the two groups (intervention vs. control), will receive a Participant ID with which the questionnaires are completed, and with which the serum 25(OH)D are transferred from DVU to the TU/e research team. Access to the key/link between the assigned number/code and names are saved in a secured local environment at the TU/e. An independent researcher at the Human-Technology department will pseudonymize (double code) the data received from DVU and obtained from the online questionnaires before sending these to the data-analyst. The double coding key will also be saved in a secured local environment at the TU/e. Data is not shared with, or stored at Signify. Analyses and reports are only on the group level (aggregated data) and not on the individual level. To protect privacy, coded data related to the pilot will be stored locally at the TU/e local server encrypted with a password. Data will be stored for a minimum of 15 year.

In order to prevent unnecessary data collection and unnecessary sharing the following actions are in place. For the blood sampling the participants will be in contact only and directly with medical partner DVU, who will do the blood sampling and the blood sample analysis. After 5 days of the analysis the remaining blood samples will be destroyed as medical waste. The laboratories will share the coded analysis results with TU/e. The independent researcher at TU/e pseudonymizes (double codes) the incoming data before sending the data set to the responsible investigator who will do the analyses. In this way, the researcher doing the analyses cannot identify individual participants in the final dataset. Participants can change their mind about including their data in the analysis until 24 hours after the final measurement day of the study.

All other data (questionnaire data) are stored locally at the TU/e server (encrypted, coded data). Access to the data will be limited to the responsible researchers. Data will additionally be protected by a strong password generated by a Microsoft approved password generator.

11.2 Monitoring and Quality Assurance

The WMO requires on-site monitoring for drug related research conform the ICH-GCP regulation (2005/28 EG). However, in case the clinical trial is not related to drugs and still WMO obligatory monitoring is not legally required. The intervention of the proposed clinical trial is a lighting intervention and not a drug. Therefore, a request for exemption is made.

11.3 Amendments

Amendments are changes made to the research after a favourable opinion by the accredited METC has been given. All amendments will be notified to the METC that gave a favourable opinion.

11.4 Annual progress report

An annual progress report will be sent to the METC. Since the pilot study is estimated to run from September till January including data gathering, analysis and reporting, the team will supply the end report two months after the end of the study the latest.

11.5 Temporary halt and (prematurely) end of study report

The METC will be notified immediately of a temporary halt of the study, including the reason of such an action.

11.6 Public disclosure and publication policy

The first aim of this pilot study is to explore the effectiveness of adding artificial UVB in general lighting solutions to sustain sufficient serum 25(OH)D in office workers. Secondary aims encompass effects on sleep quality and fatigue. The research team at TU/e will therefore report the results of this field study by means of an internal report and presentation for Signify. In addition, a publication in a peer-reviewed journal about the objective parameter (serum 25(OH)D) and correlational data about the questionnaires (sleep and fatigue) will be considered.

12. STRUCTURED RISK ANALYSIS

To ensure the newly developed prototype is safe to use in the clinical trial, a risk assessment has been conducted to identify potential risks and to take appropriate actions to alleviate these identified risks by (1) inherent safety by design, (2) protective measures in the medical device itself or in manufacturing process and (3) information for safe use. The risk assessment team included technicians, system architects, quality control people, photobiological safety experts both internally and externally, external UV-B expert, approbation specialists both internally and externally, human subject study experts and clinicians.

The personal desk-based lighting device carries a CE mark with document no FS484F UV-B DoC (*D6_8 CE Declaration of Conformity (FS484F UVB DoC*)

12.1 Identified risks

12.1.1 Mechanical, thermal, and electrical

Since the newly-developed luminaire prototype is based upon an existing commercially available architecture, the potential effects of failure in relation to the mechanical and electrical requirements were focused on those parts that were modified i.e. the head of the luminaire that could potentially change

- the mechanical stability
- thermal properties due to the change in LEB boards,
- material stability due to the UV-B LED
- electrical properties
- the stability of the diffuser window
- driver properties

Additional mechanical, thermal and electrical measurements were conducted to identify the potential risks severity and occurrence. The learnings were translated into construction improvements (safety by design) via protective measures in the device itself or prevention guidelines in e.g. the manual, participant information and research protocol (information for safety). Table 4 gives an overview of the risk analysis and mitigation. For more details we refer to *D1 Investigators brochure and risk analysis_v2*.

Table 4 Summary of the main identified risks, associated risk mitigation measures and remaining residual risk.

Overview risk analysis and mitigation					
Risk category	Item	Taken measure	Overall residual risk		
		inherent safety by design	occurunce of failure	severity	classification
Mechanical, thermal and electrical	mechanical stability	*weight delta is less then 1 %	1	1	Negligible
	thermal properties	*max operating T is 31°C @ 20°C; far below max touch temperature of the housing and far below max operating temperature of the components	1	1	Negligible
	material stability	*an UV resistant double isolated wiring (confirm IEC60598) is used	1	1	Negligible
	EMC properties	*electrical class 1	1	1	Negligible
	stability diffuser window	*the diffuser window is fixed by mechanical design tightening the diffuser between two plates<br>* Additional support by chicken wire	3	3	Tolerable
	drivers	*maximum current is fixed to prevent an over drive of the UV-B LED<br>*maximum current can not be changed by e.g. an app			Negigible
	malfunctioning of the luminaire	*prolonged lifetime test did not show any malfunctioning or stability changes	1	1	Negligible
photobiological safety	exceeding 0.7 SED	*Bluetooth controller is secured via a pin-code to prevent misuse<br>*The max UV-B light output is limited by a firmware setting in the driver to a safe level:<br>-80mA<br>-MAX DALI level of 241<br>Single failure prevention of the setting is added by means of a 100mA fast fuse in the UV-B LED circuit.<br>*RCT clock turns the device automatically on and off<br>*Prototype is equipped with switch inside the luminaire head, which will switch off the UV-B LEDs in case the luminaire head is opened.<br>personalize dlighting device	1	1	Negligible
	direct (prolonged) view to UV-B LED	*Protect installers: The prototype is equipped with a real time clock (RTC), which is setup at installation. After this setup, light will switch on at specified time stamps as agreed in the test protocol.<br>At power on, the luminaire will switch on at a dimmed white light level. UV-B will remain off. From next daily cycle schedule point, the light level will be set to the desired level. From that moment, the RTC will set the light in a daily cycle.	1	1	Negligible
	unawareness UV-B irradiance	* luminaire is supplied with warning signals<br>* User information and education is available	5	3	Tolerable
	prolonged skin exposure	* The ultra-low daily dose is five-fold lower than the guideline to prevent sunburn for skin type 1.	1	8	Tolerable

12.1.2 Photobiological safety

While potential risks related to the lighting intervention are expected to be minimal, exposure to the personal desk-based lighting intervention may result in undesirable experiences such as experienced discomfort (e.g., visual discomfort or eyestrain) or headache. Additionally, the use of the study protocol might result in distressing effects in relation to an oversensitivity to UVB, unknown at the start of the study. In that case if the participant notices a potential distressing effect, he/she can stop immediately.

Negative effects of UVB are associated to the formation of cyclobutylpyrimidine dimers in DNA contributing to the mechanism of erythema and sunburn formation. To place the UV dose applied in this study in context, the dose received throughout a full working day would be equivalent to 10 minutes in bright sunlight at lunchtime on a summer day in Northern Europe. It is clearly below the daily dose for the EU Safety Directive 2006/25/EC, and well below the erythema (sunburn) threshold for all skin types. Individuals who suffer from photosensitivity disorders, take photosensitizing medication or have had treatment for skin cancer will be excluded from the trial.

The main identified risk factors associated with these photobiological safety aspects:

- exceeding the 0.7 SED per day limit due to long office hours
- direct (prolonged) viewing of the UVB LED at short distances
- lack of awareness of UVB irradiance from the source

have been mitigated by design, protective measures and information for safe use (see table 1 and *D1 Investigators brochure and risk analysis_v2*).

As an additional measure, the newly developed luminaire is equipped with a real time clock ensuring that the exposure to ultra low doses UV-B will not exceed 8 hours and 20 minutes. After the first blood sampling has taken place, the luminaire will be powered by putting the plug in the mains socket by the research team. When the luminaire is connected to power it will start with dimmed white light and no UVB. A small amount of white light is emitted to indicate that the luminaire is ON. At noon or when powered after 13.00 hr, the next morning, the luminaire will switch ON (full brightness and UVB) according the preset schedule. at 8.50 hr. At 17.10 hr both the white and UV-B light will turn off. This behavior will also occur in case of a power cycle / disturbance in power. When the luminaire remains plugged, the luminaire will automatically switch ON (full brightness and UVB) at 8.50 hr. At 17.10 hr both the white and UV-B light will turn off. This will be repeated for all days during the study period as long as the luminaire is powered.

The contributary risk of developing skin cancer by exposure to ultra low doses of artificial UVB during a 2-month period is considered to be very low:

- The ultra-low daily dose is five-fold lower than the guideline to prevent sunburn for skin type 1.
- The recommendation of the KWF for required healthy sun exposure is in line with what is intended in this study.
- The potential protective effects of vitamin D to attenuate inflammation from sunburn

For more details see appendix III in *D1 Investigators brochure and risk analysis_v2*.

In summary, if the application precautions are considered to make the luminaire safe for the intended application, as described in the photobiological safety photobiological hazard (European directive 2006/25/EC), and the device can be regarded safe to use under application conditions according to IEC 62471 .

(supporting documents *D6_1 Analysis of Signify Luminaires_Final, D6_7 Manual-Installer v2.0, E4 manual user*).

12.2 Application precautions

The luminaire is calibrated at safe light output values when used as intended (at a distance of >80 cm). Respecting the indicated minimum application distances (up to 80 cm) keeps the Actinic UV exposure within Risk Group Exempt limits, which allows for a continuous exposure of 30000 sec per day (8 hrs and 20 minutes). Being at a closer distance (up to 35 cm) to the luminaire would allow for a continuous exposure of 10000 sec per day (2 hrs. and 45 minutes). At distances closer (looking and staring into the luminaire at close distance) than the indicated 35 cm, a continuous exposure of the luminaire of 1000 sec per day is allowed (15 minutes), but this should be avoided.

Prior to placement the general desk illumination should be measured to check compliancy with local home office illumination. The lighting units are internally set by the manufacturer to come on at 08:50 hr and go off at 17:10 hr when powered, restricting both the lighting and the UV supplementation to these hours of the day, so that a daily dose may not be exceeded. Controls are only accessible to trained personnel. The UV radiation dose will be monitored throughout the trial by trained personnel to ensure that the UV output of the units remains constant (see *D6_7 Installer manual v2.0*).

As extra precaution, the UV-B irradiance of the lamp will be regularly checked during the study period by the technically trained person (see *D6_7 Installer manual v2.0*):

- Via solar meter model 6.5 (UV) at 20 cm of the head of the luminaire. UVB irradiance levels on a weekly basis to ensure a consistent dose during the pilot study monitored by a point measurement
- The relative stability will also be monitored through a continuous daily dose measurement is conducted by placing wear shade dosimeters at the desk. The watch is placed a charger.

The desk-based intervention device is labelled with caution labels and a warning not to stare directly into the luminaire. The additional white lighting provides for an additional cue to the users that UVB is irradiated. To ensure proper usage in the application and operating of this engineering sample, participants are asked to adhere to the following guidelines (see *E4 Manual user and* ***Figure 5***):

- You do not have to turn on or off the luminaire. This will be conducted automatically.
- Please prevent direct view to the luminaire head
- Please, prevent prolonged exposure to distances shorter than 80 cm.
- Your desk has been taped to indicate the area of sufficient user's UV-B exposure. Please, try to work as much as possible in this area to receive the intended safe dose of UVB irradiation.
- Please, indicate to others in the home office, not participating the test, to be placed at a minimal distance of 1.6m to the luminaire.
- Please, unpower the device if you experience skin redness or eye strain and please report the event.
- Any damage or unexpected behavior should be reported directly

Co-workers, not participating the test, should be placed at a minimal distance of 1.6m to the luminaire.

12.3 Risk - Benefit Analysis

The device can be regarded safe to use under application conditions according to IEC 62471 and as such the potential risks related to the lighting intervention are expected to be minimal, exposure to the personal desk-based lighting intervention may result in undesirable experiences such as experienced discomfort (e.g., visual discomfort or eyestrain) or headache. Additionally, the use of the study protocol might result in distressing effects in relation to an oversensitivity to UVB, unknown at the start of the study. In that case if the participant notices a potential distressing effect, he/she can stop immediately.

On the other hand, UVB photons penetrating human skin are responsible for its conversion into the active form of vitamin D3. The most well-known function of vitamin D3 is its role in the development of strong bones and prevention of rickets. However, the vitamin D3 hormone is a very important and versatile molecule with a much larger impact on the human body then only bone strength: it is also involved in a very large number of genes in the human genome, and hence many tissues and cells. The last years, more and more attention is payed to vitamin D3 deficiency and the possible correlations with a wide number of health issues, like the immune system, autism, ADHD, coronary heart diseases, diabetes, several cancers, neuropsychiatric diseases (Alzheimer, dementia), infectious diseases, respiratory tract infections (influenza), cognitive functioning, and autoimmune diseases, where the vitamin D3 active metabolite 1,25(OH)2D3 , is recognized as a critical hormone regulating cell growth

and modulating the immune system. Therefore, expected benefits (particularly in intervention group) are maintenance of summer vitamin D3 levels and through that an enhanced sense-of-wellbeing.

As such the proposed unobtrusive, safe solution might be an effective tool in maintaining summer vitamin D levels in autumn/winter and through that participants may benefit from a stronger immune system, and an enhanced sense-of-wellbeing.

12.4 CE-mark

The lighting device carries a CE mark with document no FS484F UV-B DoC with description “Portable general purpose luminaire with LED modules, with secondary function of UV-B radiation for experimental purposes”. The requirements for CE (see figure 8) relate to

- Low Voltage Directive (Electrical, thermal, and mechanical safety according to luminaire harmonized standard EN 60598), Photobiological safety according to EN 60598 supplemented by EN 60335 ‘Household and similar electrical appliances – Safety’ and NEN3140
- Electromagnetic compliancy (EMC)
- Restriction of Hazardous Substances (ROHS 2011/65/EU
- IEC62471(Photobiological safety for lamps and lamp systems)

Low Voltage Directive (LVD), 2014/35/EU

- EN 60598-1:2015 + AC:2015 + AC:2018
- EN 60598-2-1:1989
- EN 60598-2-2:2012
- EN 60598-2-4: 1997
- EN 62471:2008
- IEC 62778:2014 (Second Edition)
- EN 62493:2015

Electromagnetic compatibility Directive (EMC), 2014/30/EU

- EN 55015: 2013-08: A1: 2015-05 / CISPR 15: 2013-04: A1: 2015-03
- EN 61547: 2009-08 / IEC 61547: 2009-06
- EN 61000-3-2: 2014-08 / IEC 61000-3-2: 2014-05
- EN 61000-3-3: 2013-08 / IEC 61000-3-3: 2013-05

EcoDesign requirements for energy-related products Directive (ErP), 2009/125/EC and applicable Implementing Measures

- 1194/2012
- Regulation EU/2019/2020 Article 7

Restriction of the use of certain Hazardous Substances in electrical and electronic equipment Directive (RoHS), 2011/65/EU

- EN 50581:2012

Figure 8: CE-mark information

13. REFERENCES

[1] Klepeis, N. E., Nelson, W. C., Ott, W. R., Robinson, J. P., Tsang, A. M., Switzer, P., ... & Engelmann, W. H. (2001). The National Human Activity Pattern Survey (NHAPS): a resource for assessing exposure to environmental pollutants. *Journal of Exposure Science & Environmental Epidemiology*, *11*(3), 231-252.

[2] Cajochen, C. (2007). Alerting effects of light. *Sleep Medicine Reviews, 11*(6), 453-464.

[3] Chellappa, S. L., Gordijn, M. C. M., & Cajochen, C. (2011). Can light make us bright? Effects of light on cognition and sleep. *Progress in Brain Research, 190*, 119-133.

[4]. Berson, D. M., Dunn, F. A., & Takao, M. (2002). Phototransduction by retinal ganglion cells that set the circadian clock. *Science, 295*(5557), 1070–1073.

[5] Freedman, M. S., Lucas, R. J., Soni, B., von Schantz, M., Muñoz, M., David-Gray, Z., & Foster, R. (1999). Regulation of mammalian circadian behavior by non-rod, non-cone, ocular photoreceptors. *Science, 284*(5413), 502–504.

[6] Provencio, I., Rodriguez, I. R., Jiang, G., Hayes, W. P., Moreira, E. F., & Rollag, M. D. (2000). A novel human opsin in the inner retina. *Journal of Neuroscience, 20*(2), 600–605.

[7] Lucas, R.J., Freedman, M.S., Muñoz, M., Garcia-Fernández, J.-M., and Foster, R.G. (1999). Regulation of the mammalian pineal by non-rod, non-cone, ocular photoreceptors. *Science 284*, 505–507.

[8] Thapan k, Arendt, J.; and Skene D.J. (2001) An action spectrum for melatonin suppression: evidence for a novel non-rod, non-cone photoreceptor system in humans. *J. Physiol-London 535*, 261-267.

[9] Brainard, G.C.; Hanifin J.P.; Greeson J.M., Byrne B., Glickman G., Gerner, E, and Rollag, M.D. (2001). Action spectrum for melatonin rewgulation in humans: evidence for a novel circadian photoreceptor. *The journal of neuroscience, 21,* 6405-6412.

[10] Martineau, A.R.; Jollife, D.A.; Hooperm R.L.; Greenberg, L; Aloia, J.F.; Bergman, P; Dubnov-Raz, G; Esposito, S.; Ganmaa, Davaasambuu; Ginde, G.A.; Goodall, E.C.; Grant, G.C.; Griffiths, C.J.; Janssens, W.; Laaksi, I.; Manaseki-Holland, S.; Mauger, D.; Murdoch, D.R.; Neale, R.; Rees, J.R.; Simpson, S.; Stelmach, I.; Kumar, G.T.; Urashima, M.; Camargo, C.A. (2017). Vitamin D supplementation to prevent acute respiratory tract infections: review and meta-analysis of individual participant data. *BMJ, 356*6583.

[11] Holick, M. F. (2008). The vitamin D deficiency pandemic and consequences for nonskeletal health: mechanisms of action. Molecular aspects of medicine, 29(6), 361-368.

[12] Kochevar, I. E. (1992). Acute effects of ultraviolet radiation on skin. *Biologic effects of light. Berlin, New York: Walter de Gruyter & Co,* 3.

[13] Hess, A. F., & Unger, L. J. (1921). The cure of infantile rickets by artificial light and by sunlight. *Proceedings of the Society for Experimental Biology and Medicine, 18*(8), 298-298

[14] Pludowski, P., Holick, M. F., Pilz, S., Wagner, C. L., Hollis, B. W., Grant, W. B., ... & Soni, M. (2013). Vitamin D effects on musculoskeletal health, immunity, autoimmunity, cardiovascular disease, cancer, fertility, pregnancy, dementia and mortality—a review of recent evidence. *Autoimmunity reviews, 12*(10), 976-989.

[15] Eyles, D. W., Burne, T. H., & McGrath, J. J. (2013). Vitamin D, effects on brain development, adult brain function and the links between low levels of vitamin D and neuropsychiatric disease. *Frontiers in neuroendocrinology, 34*(1), 47-64.

[16] Baeke, F., Takiishi, T., Korf, H., Gysemans, C., & Mathieu, C. (2010). Vitamin D: modulator of the immune system. *Current opinion in pharmacology, 10*(4), 482-496.

[17] Patrick, R. P., & Ames, B. N. (2014). Vitamin D3 hormone regulates serotonin synthesis. Part 1: relevance for autism. *The FASEB Journal, 28*(6), 2398–2413.

[18] Gong, Q. H., Li, S. X., Li, H., Chen, Q., Li, X. Y., & Xu, G. Z. (2018). 25-Hydroxyvitamin D Status and its association with sleep duration in chinese schoolchildren. Nutrients, 10(8), 1013.

[19] Kim, J. H., Chang, J. H., Kim, D. Y., & Kang, J. W. (2014). Association Between Self-Reported Sleep Duration and Serum Vitamin D Level in Elderly Korean Adults. Journal of the American Geriatrics Society, 62(12), 2327-2332.

[20] Jung, Y. S., Chae, C. H., Kim, Y. O., Son, J. S., Kim, C. W., Park, H. O., ... & Kwak, H. S. (2017). The relationship between serum vitamin D levels and sleep quality in fixed day indoor field workers in the electronics manufacturing industry in Korea. Annals of occupational and environmental medicine, 29(1), 25.

[21] Nakamura, K., Hui, S. P., Ukawa, S., Okada, E., Nakagawa, T., Okabe, H., ... & Tamakoshi, A. (2019). Serum 25-hydroxyvitamin D3 levels and poor sleep quality in a Japanese population: the DOSANCO Health Study. Sleep medicine, 57, 135-140.

[22] Roy, S., Sherman, A., Monari-Sparks, M. J., Schweiker, O., & Hunter, K. (2014). Correction of low vitamin D improves fatigue: effect of correction of low vitamin D in fatigue study (EViDiF Study). North American journal of medical sciences, 6(8), 396.

[23] Nowak, A., Boesch, L., Andres, E., Battegay, E., Hornemann, T., Schmid, C., ... & Krayenbuehl, P. A. (2016). Effect of vitamin D3 on self-perceived fatigue: A double-blind randomized placebo-controlled trial. Medicine, 95(52).

[24] Holick, M. F., & Chen, T. C. (2008). Vitamin D deficiency: a worldwide problem with health consequences. *The American journal of clinical nutrition, 87*(4), 1080S-1086S.

[25] Holick, M. F. (2017). The vitamin D deficiency pandemic: approaches for diagnosis, treatment and prevention. Reviews in Endocrine and Metabolic Disorders, 18(2), 153-165.

[26] Bjørklund, G. (2016). Vitamin D deficiency: a global health problem. Peertechz J *Environ Sci Toxicol 1,* (1), 23, 24.

[27] Palacios, C., & Gonzalez, L. (2014). Is vitamin D deficiency a major global public health problem?. *The Journal of steroid biochemistry and molecular biology, 144,* 138-145.

[28] Webb, A. R. (2006). Who, what, where and when—influences on cutaneous vitamin D synthesis. *Progress in biophysics and molecular biology, 92*(1), 17-25.

[29] Troesch, B., Hoeft, B., McBurney, M., Eggersdorfer, M., & Weber, P. (2012). Dietary surveys indicate vitamin intakes below recommendations are common in representative Western countries. *British Journal of Nutrition, 108*(4), 692-698.

[30] Lamberg-Allardt, C. (2006). Vitamin D in foods and as supplements. *Progress in biophysics and molecular biology, 92*(1), 33-38.

[31] Holick, M. F. (2008). Sunlight, UV-radiation, vitamin D and skin cancer: how much sunlight do we need?. In *Sunlight, vitamin D and skin cancer* (pp. 1-15). Springer, New York, NY.

[32] Tuckey, R. C., Cheng, C. Y., & Slominski, A. T. (2019). The serum vitamin D metabolome: what we know and what is still to discover. *The Journal of steroid biochemistry and molecular biology, 186*, 4-21.

[33] Grigalavicius, M., Moan, J., Dahlback, A., & Juzeniene, A. (2015). Vitamin D and ultraviolet phototherapy in Caucasians. *Journal of Photochemistry and Photobiology B: Biology, 147*, 69-74.

[34] Bogh, M. K. B., Gullstrand, J., Svensson, A., Ljunggren, B., & Dorkhan, M. (2012). Narrowband ultraviolet B three times per week is more effective in treating vitamin D deficiency than 1600 IU oral vitamin D3 per day: a randomized clinical trial. *British Journal of Dermatology, 167*(3), 625-630. [18]

[35] Biersack, M. G., Hajdukiewicz, M., Uebelhack, R., Franke, L., Piazena, H., Klaus, P., ... & Detert, J. (2016). Sustained increase of 25-hydroxyvitamin D levels in healthy young women during wintertime after three suberythemal UV irradiations—the MUVY pilot study. *PloS one, 11*(7).

[36] de Gruijl, F. R., & Pavel, S. (2012). The effects of a mid-winter 8-week course of sub-sunburn sunbed exposures on tanning, vitamin D status and colds. Photochemical & *Photobiological Sciences, 11*(12), 1848-1854.

[37] Orlova, T., Moan, J., Lagunova, Z., Aksnes, L., Terenetskaya, I., & Juzeniene, A. (2013). Increase in serum 25-hydroxyvitamin-D3 in humans after sunbed exposures compared to previtamin D3 synthesis in vitro. *Journal of Photochemistry and Photobiology B: Biology, 122*, 32-36.

[38] Jager, N., Schoepe, J., Wagenpfeil, S., Bocionek, P., Saternus, R., Vogt, T., & Reichrath, J. (2018). The impact of UV-dose, body surface area exposed and other factors on cutaneous vitamin D synthesis measured as serum 25 (OH) D concentration: systematic review and meta-analysis. *Anticancer research, 38*(2), 1165-1171.

[39] Webb, A. R., Kline, L., & Holick, M. F. (1988). Influence of season and latitude on the cutaneous synthesis of vitamin D3: exposure to winter sunlight in Boston and Edmonton will not promote vitamin D3 synthesis in human skin. *The journal of clinical endocrinology & metabolism, 67*(2), 373-378.

[40] Cannell, J. J., Vieth, R., Umhau, J. C., Holick, M. F., Grant, W. B., Madronich, S., ... & Giovannucci, E. (2006). Epidemic influenza and vitamin D. *Epidemiology & Infection, 134*(6), 1129-1140.

[41] Holick, M. F., & Chen, T. C. (2008). Vitamin D deficiency: a worldwide problem with health consequences. *The American journal of clinical nutrition, 87*(4), 1080S-1086S.

[42] Diffey, B. L. (2013). Modelling vitamin D status due to oral intake and sun exposure in an adult British population. *British journal of nutrition, 110*(3), 569-577.

[43] Beurskens, A. J., Bültmann, U., Kant, I., Vercoulen, J. H., Bleijenberg, G., & Swaen, G. M. (2000). Fatigue among working people: validity of a questionnaire measure. *Occupational and environmental medicine, 57*(5), 353-357.

[44] Carney, C. E., Buysse, D. J., Ancoli-Israel, S., Edinger, J. D., Krystal, A. D., Lichstein, K. L., & Morin, C. M. (2012). The consensus sleep diary: standardizing prospective sleep self-monitoring. *Sleep, 35*(2), 287-302.

[45] Roenneberg, T., Wirz-Justice, A., & Merrow, M. (2003). Life between clocks: daily temporal patterns of human chronotypes. *Journal of biological rhythms, 18*(1), 80-90.

[46] Hedlund, L., Brekke, H. K., Brembeck, P., & Augustin, H. (2014). A short questionnaire for assessment of dietary vitamin D intake. *European Journal of Nutrition & Food Safety*, 150-156.
