# Supplementary material for: Using a low-dose ultraviolet-B lighting solution during working hours: An explorative investigation towards the effectivity in maintaining healthy vitamin D levels
Source: PLoS One. 2023 Mar 31;18(3):e0283176. doi: 10.1371/journal.pone.0283176 (PMC10065255; doi:10.1371/journal.pone.0283176)
Supplement: Attachment — Submitted filename: Response to Reviewers.pdf [file pone.0283176.s009.pdf]

Response to reviewers

We would like to thank the reviewers for their feedback on our manuscript. In the revised manuscript we have addressed their comments and marked all changes. Below, we have provided a point-by-point response to the reviewers' comments. We hope that the changes in the manuscript address the points that were raised to the reviewers' satisfaction.

Reviewer #2:

The reviewer wrote: "Overall, I found this study as a sound and well written manuscript, however, I am not sure if it is adding much to the current knowledge. Also, the experiments design could have been improved by adding different UV light types/doses/durations."

We would like to thank the reviewer for his/her comment. To our knowledge, this is the first study which investigates the effectiveness of a daily low-dose and low body surface area UVB exposure intervention in the home/office setting to sustain healthy serum 25(OH)D during the winter period. As explained in the introduction, we think that this could offer an unobtrusive, accessible, and safe solution in the future to improve serum 25(OH)D year-round with potential secondary health benefits, also compared to oral supplementation only [1]. We feel that the recommendations in the discussion section (e.g., regarding dose, exposed body surface area, and duration of use) are especially relevant for further development and testing of these types of devices for use in the home and/or office setting. We agree that, based on the current findings, new (types of) lamps should be developed and investigated in the future that can improve effectiveness for sustaining healthy serum 25(OH)D in winter. We addressed this in the discussion section, lines 442-469 in 'Revised Manuscript with Track Changes'.

1. Tuckey RC, Cheng CYS, Slominski AT. The serum vitamin D metabolome: what we know and what is still to discover. J Steroid Biochem Mol Biol. Elsevier; 2018;186:4–21.

Reviewer #3:

The reviewer wrote: "Dear authors thanks for submitting this work to this journal but please make the attached required correction for your paper to be sound for publication on this journal and follow the required instructions"

We thank the reviewer for the feedback on our manuscript. A first important remark concerned the necessity for the control group to receive a placebo treatment. We agree with the reviewer that this would be preferable, but due to pragmatic reasons (the availability of lamps) this was not possible for the current study. However, we would like to point out that for our main study objective (effects of receiving extra UVB exposure in winter on serum 25(OH)D), the chance of a placebo effect is highly unlikely as vitamin D synthesis in the skin is purely photon-driven. Moreover, we checked and controlled for potential behavioral changes in either of the groups that may have affected serum 25(OH)D (i.e., vitamin D related food intake and time spent outside in daylight). Even though during the study period (winter) at our latitude it was not expected that extra time spent outside would affect serum 25(OH)D

[2], we still included cumulative time spent outside in daylight as a control variable in our analysis. This confirmed our expectation, as it did not significantly affect 25(OH)D levels between January and March in our study sample. Furthermore, vitamin D related food intake was not significantly different between the two groups, and the contribution of vitamin D intake via only food intake on serum 25(OH)D is very limited [3, 4]. In addition, we instructed all participants at the beginning and during the course of the study not to start taking vitamin D supplements. We further elaborated on these points in the discussion section (line 481 – 492 in 'Revised Manuscript with Track Changes'). One participant in the control group was taking a daily multivitamin which contained a low vitamin D dose (the participant found this out after the study had already started). Therefore, we decided to keep this participant in the study and perform the main analysis with and without this participant. The results were not affected by this (see lines 341-342 in 'Revised Manuscript with Track Changes'). For the subjective indicators (i.e., sleep and fatigue), on the other hand, a placebo treatment (lamp with the same visible light spectrum without additional UVB) would be necessary for testing effects in the future (addressed in the discussion section, line 492-497 in 'Revised Manuscript with Track Changes'). Therefore, we only investigated relationships between changes in serum 25(OH)D and subjective indicators over time across all study participants, instead of analyzing the effect of the intervention on these subjective indicators.

2. Webb AR. Who, what, where and when—influences on cutaneous vitamin D synthesis. Prog Biophys Mol Biol. 2006;92(1):17–25.
3. Lamberg-Allardt C. Vitamin D in foods and as supplements. Prog Biophys Mol Biol. 2006;92(1):33–8.
4. Troesch B, Hoeft B, McBurney M, Eggersdorfer M, Weber P. Dietary surveys indicate vitamin intakes below recommendations are common in representative Western countries. Br J Nutr. Cambridge University Press; 2012;108(4):692–8.

In the method section, the reviewer wrote: "How was sample size determined? add the calculation method" and "What was the method used to generate the random allocation sequence. Explain the type of randomization"

We rearranged section 2.2. and added the necessary information. We addressed the sample size calculation program, input and output here. The full data, visualizations and explanation regarding the calculation method are available in the study protocol. The link to this document was also added in this section. Moreover, we added information about the generation of the random allocation sequence.

The reviewer also wrote: "Intervention at home may affect the results as participants may not follow the instructions perfectly"

We agree that adherence to the study protocol could not be controlled entirely, this limitation is inherent to field studies. We did, however, ask participants to complete a daily diary in which they reported the number of hours worked in their home office (where the lamp was placed, and automatically switched on between 8:50 and 17:10). Response rates on this daily diary were very high (>98%; see results section). Nevertheless, we addressed this potential limitation further in the discussion section (line 451-457), suggesting a point for improvement in future studies with a similar set-up.

We adjusted each of the other points suggested by the reviewer, these changes can be found in the file 'Revised Manuscript with Track Changes'.
