# Supplementary material for: Using a low-dose ultraviolet-B lighting solution during working hours: An explorative investigation towards the effectivity in maintaining healthy vitamin D levels
Source: PLoS One. 2023 Mar 31;18(3):e0283176. doi: 10.1371/journal.pone.0283176 (PMC10065255; doi:10.1371/journal.pone.0283176)
Supplement: Attachment — Submitted filename: Response to Reviewers.pdf [file pone.0283176.s011.pdf]

We thank the reviewers for the second round of feedback on our manuscript. In the revised manuscript we have addressed the comments and marked all changes. Below, we have provided a point-by-point response to the reviewers' comments. We hope that the changes in the manuscript address the points that were raised to the reviewers' satisfaction.

Reviewer #3:

The reviewer wrote:
"Sample size calculation revealed 37 participants so you should enroll equal or more than to get sound results and allow generalization so you should justify this"

We would like to point out that the sample size calculation resulted in 34 participants (not 37, see study protocol page 16). During the participant recruitment phase, 35 participants were recruited. From these, 29 returned the signed informed consent form (see Figure 1 of the manuscript). Due to the predetermined study period timing which should be in winter (i.e., January to March for all participants) and practical limitations (i.e., only 14 UVB-containing lamps were manufactured), it was decided to start the study with 29 participants who gave written consent. We further explained these practical considerations in the revised manuscript (see lines 115-121 in 'Revised Manuscript with Track Changes'). In addition, the potential main reasons for not finding any significant effects of the intervention in the current study were further explained in the discussion section (i.e., half of the expected exposure time and a small body surface area exposure due to angle of the light exposure). Three extra participants in each group would yield a detection of only a slightly smaller effect in the current study (effect size *d* of 1.05 instead of 1.14, assuming a power of .90 and 17 participants per group). The actual effect to be detected in the current study – if there was any – was substantially smaller than expected due to less than expected UVB exposure.

The reviewer wrote:
"These parameters not clear and what SD mean is it standard deviation and why you added it?"

mW_A/m^2 is the measurement unit of Actinic weighted UVB irradiance (the sub A stands for Actinic). The CIE effective UV threshold in commercial lamps should be limited to < 30 J_A/m^2 per 8 hours within a time span of 24 hours, which results in an upper limit for actinic UV irradiance of less than 1 mW_A/m^2, which was the case for the devices we used (i.e., ~ 0.76 mW_A/m^2). This was also explained at the beginning of this section (lines 160-167 in the revised manuscript) with reference to reference number 26 (where this is further explained). SD indeed stands for standard deviation, we added these SD's because there was a slight variation in actinic UV weighted UVB irradiance between the 14 lamps used.

Reviewer #4

The reviewer wrote:

"In the limitations discussion, please include some discussion of the issues around inability to perform randomization for all subjects. While it is reasonable that there are few issues with this, it is up to the authors to make the case --- and, it cannot be said definitively that this did not play a role in the results."

We thank the reviewer for this suggestion and agree that randomization for all subjects would have been preferred. We expanded on this limitation to the discussion section (see lines 546-550 in 'Revised Manuscript with Track Changes').

The reviewer wrote:
"In Lines 235-236 the authors describe a slightly troubling methodology where data are thrown out if they are "outliers". The first part of the results section should describe all such data quality issues. How many data were discarded? Could these have important effects on the conclusions?"

and

"In general, it is very poor practice to discard data and then present results based on the "cleaned" data alone. It is better practice to present the analysis on the actual data, then present an alternative analysis on the "cleaned" data (cf. per protocol versus intent-to-treat)."

We would like to clarify that in the current analyses, we included all data, except for the explorative analyses on midsleep timing and sleep duration (section 3.5.), for which there were three illogical values (due to participant reporting) which could not be translated into a time-value (see also Table 2 in the results section, where the *N* was given added along with the mean values where this was deviant from 14). There were no outliers > 3 SD's in the variables used for the main analyses, so no observations were removed. We clarified this in the beginning of the results section (lines 297-301 in 'Revised Manuscript with Track Changes').

The reviewer wrote:
"Rather than providing demographics in the Methods section, provide a standard "Table 1" in the Results section that gives demographic and baseline characteristics for the study population by arm and overall."

We added this information at the beginning of the Results section (see Table 2), instead of in the Methods section.

The reviewer wrote:
“For section 3.4 and 3.5, please provide plots of the data.
Also, provide the same analysis for the control group as was provided for the intervention group. Otherwise, it makes it seem as though the authors do not wish to make the comparison with the control group. In the Study Protocol, the authors plan to use linear mixed models to perform these exploratory analyses. This pre-planned analysis should be done and reported, if only briefly. If it is too lengthy it can be included as a Supplementary analysis. Figures analogous to Figure 2 should also be created for these analyses.”

We added plots of the analyses of section 3.4 in the Results section (see Figure 4). We also ran the correlational analyses in section 3.4 for the control group. However, it should be noted that there is no real cumulative lamp exposure in the control group as this group did not receive a lamp at their work desk. Thus, we correlated cumulative desk time to change in serum 25(OH)D from baseline to Week 4 and baseline to Week 8, but only in the intervention group this was equivalent to actual cumulative UVB exposure.
With regard to section 3.5 it should be noted that it was never our intention to make direct comparisons between the intervention and the control group for the subjective outcome measures due to the high chance of a placebo effect because study conditions were not blinded. Participants in the intervention group knew that they were exposed to additional UVB as they received a lamp, while participants in the control group did not receive a lamp and knew they were not exposed to additional UVB. Nevertheless, for visual inspection purposes we added plots of the associations between 25(OH)D and the subjective outcome measures for both conditions together with the output of the exploratory linear mixed model analyses as described in the study protocol (see Supporting Information Tables S1 – S4, and Figure S1; and lines 439 – 445 in ‘Revised Manuscript with Track Changes’).

The reviewer wrote:
“In the Conclusions please provide a quantitative estimate of the actual benefit found, along with confidence limits. It is fine that it is not statistically significant. It is also quite useful to other researchers to be able to evaluate the likely magnitude of any effects.”

We agree with the reviewer that it is important to give some quantitative estimates of the benefits found. However, we would like to refrain from reporting statistics in the Discussion/Conclusion section as this is not common practice. However, we added extra quantitative estimates in the Results section to quantify the actual benefit. First, we decided to conduct our main analyses according to the ANCOVA as specified in the study protocol (see page 35), estimating the effect of the intervention on change in serum 25(OH)D from baseline to Week 8. This analysis yields a partial eta squared effect size together with the coefficient of the effect of the intervention (see Table 5 and lines 336 – 341 in ‘Revised Manuscript with Track Changes’). In addition, we added the actual difference scores between conditions (controlled for baseline scores and time spent outside) at Week 4 and Week 8, including the 95% confidence intervals, based on the subsequent linear mixed model analysis. We also improved the figure belonging to this analysis (Figure 2) by only showing Week 4 and 8 and not baseline values. The baseline values were estimated based on the raw values, and only the values at Week 4 and 8 resulted from the LMM analysis. Last, to visualize changes over time including baseline values across all participants, we conducted a separate LMM analysis including measurement (baseline, Week 4, Week 8) to obtain all values from one model for the figure (see Figure 3).

The reviewer wrote:
"Generally, for linear mixed model analysis as in any linear model analysis, the authors should provide a brief evaluation of the aptness of the modeling. This is typically done by evaluating the residuals from the model for symmetry, independence, homoscedasticity, and normality (of less importance arguably). Note that the authors' evaluation of normality initially is a common misguided activity --- the residuals from linear model analysis are typically assumed to be normally distributed, not the actual data. Considering the structure of the authors' data will immediately make it clear why the original data do not need to be normally distributed, if the underlying linear model is correct."

We agree with the reviewer that normality of the residuals (assumption for LMM analysis) is important to inspect instead of (primarily) normality of the raw data. We checked the symmetry, independence, homoscedasticity, and normality of the residuals, and verified the aptness of the modeling in the statistical analyses section (see lines 271-286 in 'Revised Manuscript with Track Changes').

The reviewer wrote:
"The following are minor comments:

Line 112: Replace "revealed" with "required".

Line 239: Do not capitalize "Linear Mixed Model".

Line 240: Put a comma after "random intercept". The sentence is wrong otherwise.

Line 281: Do the authors mean "upper arms" here? It seems unlikely that under-arms were exposed by rolling up sleeves."

We corrected / clarified the points above in the revised manuscript.

The reviewer wrote:
"Table 4: What is the "B estimate"? Since this is derived from a linear mixed model, the authors should also provide the estimates for the random effects."

The B estimate label was changed to "Estimate (unstandardized coefficient)" (derived from the linear mixed model analysis). Random effects estimates were added to this table (Table 6 in the revised manuscript).
