# Supplementary material for: Using a low-dose ultraviolet-B lighting solution during working hours: An explorative investigation towards the effectivity in maintaining healthy vitamin D levels
Source: PLoS One. 2023 Mar 31;18(3):e0283176. doi: 10.1371/journal.pone.0283176 (PMC10065255; doi:10.1371/journal.pone.0283176)
Supplement: Attachment — Submitted filename: Manuscript_VitDStudy_revisedv2.docx [file pone.0283176.s008.docx]

**Using a low-dose UVB lighting solution during working hours: An explorative investigation towards the effectivity in maintaining healthy vitamin D levels**

L. M. Huiberts^a^, K. C. H. J. Smolders^a^, B.M.I. van der Zande^b^, R.C. Broersma^b^, & Y. A. W. de Kort^a^

a Human-Technology Interaction, School of Innovation Sciences, Eindhoven University of Technology, Eindhoven, the Netherlands

b Signify Research, Eindhoven, the Netherlands

Trial registration: <https://www.isrctn.com/ISRCTN47902923>

Corresponding author:
Laura Huiberts, Human-Technology Interaction Group, Eindhoven University of Technology, P.O. Box 513, 5600 MB Eindhoven, The Netherlands.

email: L.M.Huiberts@tue.nl

**Abstract**

This study examined whether daily safe, low-dose UVB exposure using a home-based lighting solution could maintain healthy serum 25(OH)D during winter. Twenty-eight nine-to-five indoor workers (12 male; mean age = 42.46; *SD* = 14.23) participated in this study and were allocated (via block randomization were possible) to one of the two study conditions. During an 8-week period, fourteen participants received extra UVB exposure (max 0.3 SED daily), while the fourteen participants in the control group and did not receive extra UVB exposure. Daily questionnaires were used to measure UVB exposure time, exposed body surface area (BSA), and time spent outside in daylight. Serum 25(OH)D and secondary parameters (i.e., subjective fatigue, sleep timing and quality) were investigated at baseline, Week 4, and Week 8. Serum 25(OH)D significantly declined over the 8-week study period in both groups. The combination of using a low-dose UVB exposure, a small BSA, and a lower-than-expected amount of exposure hours likely resulted in an insufficient received UVB dose to significantly improve serum 25(OH)D. Changes in serum 25(OH)D over time did not significantly correlate with changes in secondary parameters of sleep and fatigue. Future research could explore whether a longer lasting exposure period and/or using different exposure positions of the device (maximizing exposed skin surface) yields more promising results for improving serum 25(OH)D.

Keywords: UVB exposure; vitamin D; sleep; fatigue

**1. Introduction**

Vitamin D insufficiency (< 30 ng/ml; <75 nmol/L) affects more than 70% of the general population worldwide [1]. In the long term, Vitamin D insufficiency has been associated with many health consequences, such as osteoporosis, cancer, and autoimmune disorders [2,3]. In the short term, low Vitamin D levels can impede normal immune functioning, making people susceptible to develop more severe complaints from bacterial and viral infections [4]. The main route of Vitamin D synthesis is via ultraviolet B (UVB) exposure. However, due to indoor lifestyles (~70-90% of our time [5–7]), many people miss out on the benefits of UVB exposure, even in summer season [8]. Living at higher latitudes prevents effective Vitamin D synthesis between September and April, and in summer before 11:00 and after 15:00, due to the large solar zenith angle [9]. Moreover, food sources do not contain adequate Vitamin D concentrations to ensure sufficient (> 75 nmol/L) serum Vitamin D (25(OH)D) levels [10,11].

In addition to bone health and immunity, recent studies proposed underlying mechanisms explaining why daily vitamin D synthesis may also play an important role in facilitating general mental wellbeing and sleep [12], due to its role in serotonin and melatonin regulation [13,14]. Given the high prevalence of Vitamin D insufficiency, increasing Vitamin D status in the general population is expected to positively influence daytime feelings of wellbeing and sleep quality at night [12]. To that regard, ensuring sufficient vitamin D levels in the general population may not only decrease health care costs but also economic costs by increasing productivity at work and preventing sick leave [15].

Daily supplementation with Vitamin D can be a good solution to reach sufficient serum 25(OH)D. However, adherence to a Vitamin D supplementation scheme is very important to sustain sufficient levels, which may not be easy for everyone. Moreover, vitamin D synthesis via UVB exposure is expected to be more efficient in raising serum 25(OH)D compared to supplement intake [16]. Previously, an 8-week randomized controlled trial comparing short (12 minutes) UVB exposure sessions using tanning beds (three times a week) to a relatively high daily dose of Vitamin D (1000IU, 25 µg) revealed that UVB exposure was more effective in increasing serum 25(OH)D [17]. Last, UVB exposure also exclusively leads to the synthesis of different Vitamin D metabolites in the skin which all have unique contributions to health and wellbeing, such as anti-inflammatory effects [18].

Because of the above-mentioned benefits, receiving low-dose artificial UVB exposure in winter seems an unobtrusive solution to improve serum 25(OH)D, which may also yield secondary health benefits. Previous studies using UV light sources to improve 25(OH)D (controlled and uncontrolled studies) suggest that exposure to relatively low intensity narrow band UVB (i.e., significantly less UV than monthly ambient doses from sunlight) may be adequate for maintenance of healthy 25(OH)D levels throughout winter [17,19–23].

Previous dose-response study showed that a cumulative standard erythema dose (SED) of 8 delivered over a 16-week period already proved to be sufficient to sustain summer 25(OH)D levels in winter compared to receiving no extra UVB exposure [24]. However, as in most previous intervention studies, this study delivered doses infrequently (i.e., every two weeks) using a large body surface area (BSA) (i.e., 88%). Another study revealed that a cumulative dose of 13 SED received during seven subsequent days on only arms and face (~14% body area) was sufficient to realize a significant pre-post increase in serum 25(OH)D [25]. These previous studies generally used sunbed applications or (medical) treatment lamps (e.g., for psoriasis), and exposure protocols that interfere with people’s daily routines as they require visits to a hospital or clinic to receive the UVB exposure.

To date, no studies have been reported that investigated the effectiveness of prolonged low-dose UVB exposure in the home environment using a relatively small BSA on serum 25(OH)D. Therefore, we explored for the first time whether a low and safe [26] dose of UVB exposure applied in a home office lighting solution would be sufficient to maintain healthy serum 25(OH)D during winter in nine-to-five indoor workers wearing their regular clothing. Taking into account the relatively small BSA in our study (generally hands, face and neck), we hypothesized that the maximum dose of 0.3 SED a day would at least be able to reveal an attenuated decline in serum 25(OH)D during the 8-week winter period. In contrast, in the control group, a sharp decline was expected as is usually the case during the winter period in people not taking supplements [27,28]. In addition, we explored correlations between changes in serum 25(OH)D and sleep (midsleep, sleep duration and quality) and general levels of daytime fatigue over time.

**2. Materials & Methods**

*2.1. Design*

A between-subjects design was employed to investigate the effect of low-dose vs. no additional UVB exposure during working hours on serum 25(OH)D. Randomization to the study conditions was realized where possible (see section *2.2.*). UVB vs. no UVB exposure was the independent variable and serum 25(OH)D was the primary dependent variable in the study. The study was approved by the medical ethical committee of the Maxima Medical Center Veldhoven, The Netherlands. Trial registration on ISRCTN ([ISRCTN47902923](https://www.isrctn.com/ISRCTN47902923)) was carried out retrospectively so that the developed technology did not have to be shared publicly at an early stage. The full study protocol is also available from ISRCTN. Authors confirm that all ongoing and related trials for this intervention are registered.

*2.2. Participants*

All participants in this study were recruited and checked for participation eligibility by an independent recruitment organization to ensure independency of the research team. Recruitment until 28 eligible participants were enrolled in the study was conducted from November 23^rd^, 2020, through December 18^th^, 2020.

Twenty-eight nine-to-five indoor workers participated in the study, of which 14 in the intervention group (7 male; mean age = 43.3; *SD* = 16.3) and 14 in the control group (5 male; mean age = 41.6; *SD* = 12.4). One participant allocated to the control group dropped out at the beginning of the study due to job changes which resulted in too little time to participate. Figure 1 shows a complete flowchart of the participant allocation. For this study, 14 lighting devices were available in the intervention group. Therefore, two equally large groups were created to which participants were allocated. Inclusion and exclusion criteria for participation can be viewed in Table 1. Although vitamin D supplement use was an exclusion criterion, one participant in the control group indicated in the second week of the study to take vitamin D in a multivitamin (10 micrograms daily). It was decided to leave this participant in the study by asking to continue to take this supplement daily to keep conditions constant over the study period.

Participants were randomly allocated to the two study conditions – via block randomization – as much as possible (see Figure 1). However, this was not possible for several participants as they either changed in their work location or amount of working hours at home before the start of the study (n = 5), because they did not have grounded electricity outlets in their houses needed for the installation of the lamp (n = 3), or because there was insufficient space for installation of the lamp (n = 1). This information was obtained after the recruitment phase had ended, and these participants were still willing to participate in the study. To this end, nine participants were allocated to the control group before block randomization was carried out for the remainder of the participants. Subsequently, block randomization was conducted so that 14 participants were allocated to the intervention group (i.e., 14 lamps were available), and six participants were allocated to the control group. Randomization was conducted by the principal investigator (first author) of the study.

*– Insert Figure 1 about here –*

| **Inclusion criteria** | **Exclusion criteria** |
| --- | --- |
| - Age 18 or older | - Current pregnancy, breast feeding or a desire to become pregnant |
| - Fitzpatrick skin type II or III | - Having children at home aged 10 years or younger |
| - Living in/around Eindhoven | - Having malignant skin conditions in the past or currently |
| - Medically fit to work the hours as contractually agreed | - Photosensitive medical conditions or photo-sensitising drugs |
| - Desk presence (at the home office) of at least 2.5 days per week during the 8 weeks study | - Users of medicines and/or crèmes mentioning in the prescription as side effect extra sensitivity to the sun / interaction with sun exposure |
| - Finding it no problem to have blood drawn three times over a period of 8 weeks | - Planned use of sun beds, or sunbed use during the past 4 weeks |
|  | - Currently taking or planning to take oral vitamin D3 supplements or have been taking D3 supplements during the past 4 weeks |
|  | - High vitamin D levels at the start of the study (>375 nmol/L) which need medical attention [40] |

**Table 1.** In- and exclusion criteria for participation

*2.3. Setting and materials*

The study was performed in participants’ home offices as COVID-19 regulations asked people to work from home during the study period (January 2021 to March 2021 in The Netherlands). For participants in the intervention group, a lamp was installed at the home office before the start of the study. In the visual wavelength range, the lamp provided an illuminance of about 1100 lx (~ 3700 K) at 20 cm distance and about 100 lx at 80 cm distance. Additionally, the lamp emitted a continuous ultra-low and safe artificial actinic UV irradiance of less than 3.0 mW/m^2^ at 20 cm distance and 0.77 mW/m^2^ at 80 cm distance [26]. This resulted in an estimated maximal received dose of ~0.3 SED (UVI = 0.04) per 8-hour workday when seated at the desk. Participants in the intervention group and members of their household were advised to respect the indicated minimum application distance (80 cm). This distance keeps the actinic UV exposure within Risk Group Exempt limits, which allows for a continuous exposure of 30000 sec per day (8 hrs. and 20 minutes) according to the IEC62471 [26]. The UVB irradiance stability was checked by a before and after actinic UVB measurement at the Lighting Test Center Europe, Eindhoven. The equipment used for these measurements consisted of a Cary 17d monochromator and a Keithley 2000 multimeter. The scanned spectral range was 200-800 nm and measurements were executed at 80 cm distance. The averaged actinic UV weighted UVB irradiance before and after were respectively 0.76 mW_A_/m^2^ (SD = 0.04 mW_A_/m^2^ ) and 0.75 mW_A_/m^2^, (SD = 0.07 mW_A_/m^2^ ). UV Irradiance stability during the study period was checked by measuring UV-intensity in Week 1, Week 4, and Week 8 using the Shade UVR sensor at 80 and 20 cm distance from the lamp. This deviated from the original study protocol, in which weekly solar meter measurements and continuous UV measurements at the table were proposed. Three instead of weekly UV measurements were chosen to minimize the amount of visits to participants’ homes, which was strongly advised during this time of the COVID-19 pandemic in the Netherlands. In addition, only the Shade UVR sensor was used for measurements as it is much more sensitive than the solar meter. Continuous measurement at the home office table was discarded due to potential privacy issues with the Shade device, which had to be connected to participants’ personal smartphone while logging personal information. The irradiance stability throughout the study period was realized by an adjustment of the DALI settings at the end of Week 4 to correct for the slow decline in UV intensity due to the lifetime degradation of the used UVB LEDs. Participants in the control group did not receive a lamp in their home office and were therefore not exposed to extra UVB.

*2.4. Measurements*

*2.4.1. Serum vitamin D*

Blood samples were taken three times during the study period (Baseline, Week 4, and Week 8) to establish serum 25(OH)D in nmol/L. Samples were analyzed by an independent local medical laboratory (Diagnostiek Voor U) using the automated immunoassay method. Due to potential fluctuations in serum 25(OH)D over the day [29,30], participants were asked to have their blood sampled at approximately the same time of day.

*2.4.2. Sleep, Fatigue, and Vitamin D related food intake*

Sleep timing and quality, fatigue, and vitamin D related food intake were measured three times during the study period (Baseline, Week 4, and Week 8) using an online questionnaire. Sleep timing and quality were measured by integrating items from the Consensus sleep diary [31] and the Munich Chronotype Questionnaire [32], adapted for measurement over the past two weeks. Sleep duration was calculated by the difference in time between sleep onset and sleep offset, minus the total amount of minutes lying awake at night. Midsleep timing was calculated by adding half of the total sleep duration to the sleep onset time. Sleep quality was the average score of four items measured on a 5-point Likert scale. These four items probed 1) overall sleep quality; 2) easiness to fall asleep; 3) feeling refreshed at wake-up; and 4) easiness to get out of bed (Cronbach’s α = 0.79). Last, subjective fatigue was measured using the Checklist Individual Strength (CIS) [33], the sum score was used as outcome variable. Vitamin D related food intake during the past four weeks was measured using a short questionnaire previously developed by Hedlund et al. [34], including intake of (fortified) cereals.

*2.4.3. Daily diaries*

Daily diaries at the end of each workday measured time spent working at the desk (exposure duration); time spent outside (on Mondays reporting of time spent outside in the weekend was added); and exposed skin surface based on clothing (intervention group only). Participants received a daily link to this online questionnaire via e-mail between 5 and 6 PM.

*2.5. Procedure*

One month before the start of the study, participants were called by the responsible researcher to receive information about the procedure of the study and double-check the in- and exclusion criteria. Afterwards, participants were sent the study information and consent form, and returned the signed consent form to the researcher via mail when they decided to participate. During the 1.5-week period before the start of the study, participants completed the baseline questionnaire and the first blood sampling measurement. In addition, the lamps were installed in the intervention group’s homes by a technician. They received additional instructions about the lamp functionalities and behavioral guidelines during the study period. Subsequently, the 8-week study period started for both groups (January 8, 2021). Monday-Fridays, the lamps turned on automatically at 8:50 and off at 17:10 hr. In weekends, and between 17:11 and 8:49 hours the lamp was switched off (i.e., no visual light and no UVB exposure). Participants received the daily questionnaire at the end of each workday. In Week 4 and 8, the blood sampling and the main questionnaire were repeated for all participants. After Week 8, the lamps were deinstalled by the technician and participants received a financial compensation for their participation. Participants received a debriefing and overview of the main findings one month after the end of the study.

*2.6. Statistical analyses*

All statistics were performed in IBM SPSS Statistics 23.0. Before data analysis, Shapiro-Wilk tests were conducted, and skewness and kurtosis values were inspected to assess normality of the variables used in the analyses. Values more than 3 standard deviations from the mean were classified as outliers and coded as missing.

First, descriptive statistics of the baseline variables for both conditions were reported and compared between groups via independent sample T-tests. Subsequently, effects of UVB exposure on serum 25(OH)D at Week 4 and Week 8 were investigated using Linear Mixed Model analysis with participant ID as a random intercept controlling for cumulative time spent outside and baseline serum 25(OH)D. In case a significant condition * measurement (time) interaction was found (*p* < 0.05), post-hoc comparisons were conducted to investigate differences between groups at the last measurement point (Week 8), and at Week 4 to explore intermediate effects. For the intervention group only, we conducted exploratory Pearson correlation analyses investigating the association between cumulative desk presence (UVB exposure) until Week 4 and until Week 8 and respectively change in serum 25(OH)D from baseline to Week 4 and Week 8.

Last, exploratory Pearson correlations were conducted to investigate the secondary study aims, namely the relationships between changes in serum 25(OH)D and changes in sleep duration, midsleep timing, sleep quality, and general fatigue over time (Baseline to Week 8).

**3. Results**

*3.1. Response rates and descriptive statistics at baseline*

All three assessments (i.e., blood samplings and main questionnaires at baseline, Week 4, and Week 8) were 100% completed. Only two participants in the intervention group missed some of the daily questionnaires (ten of 560 daily questionnaires were missing), while three participants in the control group missed some daily questionnaires (eight of 560 daily questionnaires were missing). Table 2 shows the descriptive statistics of the variables measured at baseline. Independent sample T-tests at baseline revealed that only sleep duration on workdays significantly differed between the two groups (*p* = 0.02).

**Table 2.** Descriptive statistics of all variables measured at baseline

|  | | **Intervention group** | | | | **Control group** | | | | | |
| --- | --- | --- | --- | --- | --- | --- | --- | --- | --- | --- | --- |
|  | M | | SD | Min | Max | |  | M | SD | Min | Max |
| Serum 25(OH)D (nmol/L) | 59.00 | | 15.36 | 42 | 97 | |  | 57.79 | 19.53 | 28 | 84 |
| Fish intake^1^  (grams) | 365.00 | | 367.96 | 0 | 1200 | |  | 262.86 | 320.06 | 0 | 1200 |
| Milk intake^1^ (litres) | 1.92 | | 2.49 | 0 | 7.00 | |  | 1.83 | 2.92 | 0 | 8.40 |
| Yoghurt intake^1^ (litres) | 1.43 | | 2.15 | 0 | 7.00 | |  | 1.85 | 2.53 | 0 | 8.00 |
| Butter intake^1^ (portions on bread) | 67.00 | | 87.48 | 0 | 224 | |  | 32.71 | 45.27 | 0 | 168 |
| Sleep duration (hours) | 6.54 (*N*=12) | | 0.75 | 5.25 | 7.67 | |  | 7.59 (*N*=13) | 1.25 | 5.25 | 9.75* |
| Midsleep (hh:mm) | 03:23 (*N*=12) | | 00:45 | 02:00 | 04:30 | |  | 03:26 (*N*=13) | 00:59 | 01:38 | 04:45 |
| Sleep quality (scale 1-5) | 3.16 | | 0.86 | 1.00 | 4.50 | |  | 3.36 | 0.71 | 1.75 | 4.50 |
| Time spent outside workdays (minutes) | 66.79 | | 61.73 | 20 | 260 | |  | 87.50 | 68.80 | 10 | 250 |
| Time spent outside free days (minutes) | 84.86 | | 41.81 | 3 | 150 | |  | 139.29 | 118.69 | 20 | 500 |
| Subjective fatigue; CIS total score^2^ (range: 20-140) | 68.29 | | 24.14 | 21 | 110 | |  | 66.64 | 21.27 | 23 | 95 |

^1^ Total intake during the past four weeks

^2^ A higher score indicates more subjective fatigue

*Note.* cereal consumption was not further reported here, as only one participant in the control group ate a cereal brand with a small amount of added vitamin D.

*3.2. Descriptive statistics daily questionnaires*

On average, participants in the intervention group were exposed to the lamp for 23.67 hours per week (*SD* = 5.39; Min = 15.59; Max = 31.83). Table 3 shows the cumulative amount of exposure hours until Week 4 and Week 8. In addition, Table 3 shows the cumulative amount of time spent outside in hours per group. There was no statistically significant difference between the two groups in Week 4 (t= 1.37; *p* = 0.18) or Week 8 (t = 1.42; *p* = 0.17) in the cumulative time spent outside. The Cohen’s *d* was moderate; respectively 0.52 and 0.54. Lastly, exposed BSA was inspected in the intervention group. The hands and face (BSA ≈ 9%) were exposed in most cases (resp. 96.8% and 96.7%), while the neck area (BSA ≈ 1%) was exposed in 81.1% of the cases. The upper area of the head (BSA ≈ 1%) was additionally exposed in two persons, who had little to no hair. Finally, three of the 14 participants had their (under)arms (BSA ≈ 4%) exposed on most of the days during the study period, either by wearing a T-shirt or by rolling up their sleeves.

**Table 3.** Cumulative exposure time and time spent outside in hours over the study period

|  | | **Intervention group** | | | | **Control group** | | | | | | |
| --- | --- | --- | --- | --- | --- | --- | --- | --- | --- | --- | --- | --- |
|  | M | | SD | Min | Max | |  | M | SD | Min | Max |  |
| Lamp exposure Week 4 (midterm) | 92.30 | | 18.86 | 51.25 | 124.50 | |  | - | - | - | - |  |
| Lamp exposure Week 8 | 189.33 | | 43.13 | 124.75 | 254.63 | |  | - | - | - | - |  |
| Time spent outside  Week 4 (midterm) | 20.93 | | 8.46 | 8.25 | 39.25 | |  | 29.05 | 20.44 | 5.69 | 87.70 |  |
| Time spent outside  Week 8 | 50.06 | | 15.47 | 28.42 | 81.25 | |  | 66.08 | 39.06 | 22.86 | 179.20 |  |

*3.3. Effects of the intervention on serum 25(OH)D*

The findings of the LMM analysis investigating the effect of the intervention on serum 25(OH)D are shown in Table 4. The findings do not reveal a statistically significant effect of condition on serum 25(OH)D. There was also no statistically significant relationship between cumulative time spent outside during the past four weeks and serum 25(OH)D. As expected, there was a significant relationship between baseline serum 25(OH)D and subsequent measures of serum 25(OH)D. In addition, there was a significant effect of measurement on serum 25(OH)D indicating a significant decrease in 25(OH)D from Week 4 to Week 8. A separate LMM analysis including the interaction between condition and measurement to the model did not reveal a significantly different change in serum 25(OH)D from Week 4 to Week 8 between conditions (see Table 4). The findings of this analysis are visualized in Figure 2.

**Table 4.** Statistics Linear Mixed Model analysis; effect of intervention on serum 25(OH)D

|  | **B estimate** | **SE** | **95% CI** | **F** | **df** | **p-value** |
| --- | --- | --- | --- | --- | --- | --- |
| Condition | 1.49 | 2.64 | -3.91 – 6.89 | 0.32 | 1, 28 | 0.58 |
| Measurement | -3.72 | 1.60 | -6.95 – -0.49 | 5.43 | 1, 38 | 0.03 |
| Baseline | 0.59 | 0.07 | 0.44 – 0.75 | 63.57 | 1, 28 | <.001 |
| Time spent outside | 0.002 | 0.001 | -0.00 – 0.00 | 1.36 | 1, 37 | 0.25 |
| Condition * Measurement | -3.59 | 2.81 | -9.35 – 2.16 | 1.64 | 1, 28 | 0.21 |

*– insert Figure 2 about here –*

**Figure 2.** Average serum 25(OH)D over time in both groups, error bars represent SE’s. The study period was from January (baseline) to March (week 8)

*3.4. Correlations between exposure time and change in serum* *25(OH)D*

Two Pearson correlations were computed for the intervention group only to explore whether there were significant associations between cumulative exposure time and serum 25(OH)D in Week 4 and Week 8, respectively. The relation between cumulative exposure time until Week 4 and change in serum 25(OH)D from baseline to Week 4 showed a moderate correlation (r = 0.47) that failed to reach statistical significance (*p* = 0.09). The correlation between cumulative exposure time until Week 8 and change in serum 25(OH)D from baseline at Week 8 was low, and nonsignificant (r = 0.10; *p* = 0.75). This indicates that participants who spent more time under the lamp at the end of the study period did not achieve higher serum 25(OH)D levels compared to participants with a lower exposure time.

*3.5. Correlations between change in serum 25(OH)D and change in sleep duration, sleep quality, midsleep timing and subjective fatigue*

Pearson correlations in the full participant sample were investigated between change in serum 25(OH)D (baseline to Week 8) and change in respectively subjective measures of sleep duration, sleep quality, midsleep timing and fatigue. Table 5 shows that none of the change scores of the subjective indicators significantly correlated with change in serum 25(OH)D.

**Table 5.** Pearson correlations between change scores in serum 25(OH)D
and sleep duration, midsleep, sleep quality and subjective fatigue.

|  | Sleep duration | Midsleep | Sleep quality | Subjective Fatigue (CIS) |
| --- | --- | --- | --- | --- |
| Serum 25(OH)D | -0.15 (*p* = 0.50) | 0.00 (*p* = 0.99) | 0.16 (*p* = 0.43) | 0.01 (*p* = 0.95) |

**4. Discussion**

This study investigated whether using a low and safe dose of artificial UVB exposure applied in an office lighting solution could significantly attenuate the decline in serum 25(OH)D normally seen during the winter period in people living on the northern hemisphere. Our secondary aim was to explore whether changes in serum 25(OH)D correlated with changes in subjective sleep duration, midsleep timing, sleep quality, and general feelings of fatigue. The findings revealed that the current combination of received UVB dose and exposed BSA (~10%) in the intervention group was insufficient to attenuate a decline in serum 25(OH)D during the 8-week study period. In addition, in the current sample, there were no indications that declines in serum 25(OH)D during the winter period significantly correlated with changes in sleep duration, midsleep timing, sleep quality, and subjective fatigue.

With respect to the ineffectiveness of the intervention on changing vitamin D status, it is important to take into account the average dose received in the intervention group and compare this to previous studies using similar doses. The average weekly exposure time in the intervention group was almost 24 hours, while the maximum exposure time per week could have been approximately 42 hours. This means that the estimated average weekly UVB dose received in this sample was about 0.9 SED, and about 7 SED cumulatively. In most participants, this dose was only exposed to the face, neck, and hands (~10% BSA). A previous study investigating a BSA exposure of approximately 14% (face, hands and arms) revealed that a cumulative dose of 13 SED received during seven subsequent days was sufficient to reveal a significant increase in serum 25(OH)D [25]. Another study examined three groups of participants during a 16-week UVB exposure period in winter receiving respectively a cumulative UVB dose of 4, 8, or 16 SED, and compared these to a control group [24]. Their findings revealed that a cumulative dose of 8 SED was just sufficient to prevent the decline in serum 25(OH)D that was observed in the control group. However, there was no significant difference in serum 25(OH)D over time between the 4 SED group and the control group. It is important to note that the exposed BSA was much larger in this previous study (i.e., 88%). Both studies [24,25] included a similar number of participants in each study group as in the current study. Considering these previous findings, it is likely that the cumulative dose in combination with the relatively small exposed BSA was insufficient to create meaningful changes in serum 25(OH)D.

We also investigated correlations between cumulative exposure time and 25(OH)D levels at Week 4 and Week 8 to explore dose-dependency. While a trend towards a moderate correlation was found at Week 4, this correlation turned out to be very small at Week 8. Again, this indicates that the total dose was likely too low to realize significant changes in serum 25(OH)D, even in participants with a higher amount of exposure hours. In this study, there was too little variation in exposed BSA to make reliable correlations with changes in serum 25(OH)D levels.

One way to increase the cumulative dose is to increase the duration of study period. It should be noted that the current study started in the middle of winter, when average vitamin D levels were already relatively low compared to average levels just after summer [27,28]. It would be useful to gain insight in whether these doses could be sufficient to prevent a sharp decline in serum 25(OH)D when it is implemented directly after summer and extended towards early spring. When applied from October through March, this would yield a cumulative dose of approximately 21 SED. Moreover, the total dose could be further enhanced by also using the lighting device during weekend days and by making it portable so that it can be used at different places in the home.

Another important aspect that differentiates this study from previous studies was that the UVB exposure occurred during participants’ daily work routines. This made it hard to control participants’ distance from the luminaire and their seating position. Even though a seating position was marked on participants’ desks, and they were instructed not to move the luminaire, changes to these positions may have happened and could have impacted the total received dose. The maximum dose of 0.3 SED per day was determined based on UVB measurements using a static mannequin seated at exactly 80 cm distance from the luminaire. In real-life conditions, participants likely moved to different positions depending on the task they were performing (i.e., calling, typing, writing, reading), which may decrease the exposed BSA. This variation may have further decreased the actual received dose. Previous research revealed that the role of exposed BSA in vitamin D synthesis efficacy is especially relevant when being exposed to relatively low UVB doses [35]. At the same time, another study found that skin surface of the hands and face are most effective in synthesizing vitamin D compared to other body sites [36]. Therefore, positioning of the device and the user are important aspects to take into account in future research in order to maximize the exposed skin surface area of the face and hands.

The current findings did not reveal any significant relationships between change in serum 25(OH)D and change respectively sleep duration, midsleep timing, sleep quality, and fatigue. Likely, the current sample size was too small to reveal small to medium relationships between these variables. Previous research using larger sample sizes revealed significant associations between serum 25(OH)D and sleep indicators, usually indicating that higher vitamin D status relates to better sleep scores but the reverse has also been reported in some studies [12,37]. With respect to fatigue, intervention studies using oral supplements revealed that chronic fatigue symptoms were significantly reduced after correcting a vitamin D deficiency in people with chronic illness [38] as well as healthy people [39]. Future studies administrating a higher UVB dose, which prevents the decline in serum 25(OH)D in winter, should further investigate whether this may be beneficial for sleep and daily fatigue levels.

A limitation of the current study is that the control group did not receive a placebo intervention, which may have influenced participant behaviors in both groups differently. We do not think that this has biased the objective measure of serum 25(OH)D as this could have only been significantly impacted by supplement intake, which participants did not start taking during the study period. Moreover, food intake only has very little impact on serum 25(OH)D [10,11] and did not significantly differ between the two groups according to the questionnaires. Finally, as expected [9], cumulative time spent in natural daylight during the winter period did not significantly correlate with changes in serum 25(OH)D. Nevertheless, for further investigation of subjective health and wellbeing indicators, blinding the manipulation such that both conditions receive a lighting device with the same (visible) light spectrum is of importance.

**5. Conclusion**

The current findings revealed that a maximum low-dose UVB exposure of about 0.3 SED/day with an average weekly exposure time of 24 hours and a BSA of about ~10% is insufficient to effectively attenuate decreases in serum 25(OH)D over a period of eight weeks in winter. Future research could explore whether a longer lasting exposure period starting directly after summer until the beginning of spring yields more promising results for improving vitamin D status. In addition, future studies should focus on using different exposure positions of the device, maximizing the exposed skin surface of the hands, neck, and face. Continuation of the development and investigation of these home-based UVB lighting solutions could offer an accessible, safe, and easy-to-use method to maintain healthy vitamin D levels year-round, and potentially promote health and wellbeing in the general population.

**Conflict of Interest Statement**

This research was funded by Signify, Eindhoven, the Netherlands. Data was independently analyzed and reported by Eindhoven University of Technology.

**References**

1.	Palacios C, Gonzalez L. Is vitamin D deficiency a major global public health problem? J Steroid Biochem Mol Biol. 2014;144:138–45.

2.	Pludowski P, Holick MF, Pilz S, Wagner CL, Hollis BW, Grant WB, et al. Vitamin D effects on musculoskeletal health, immunity, autoimmunity, cardiovascular disease, cancer, fertility, pregnancy, dementia and mortality—a review of recent evidence. Autoimmun Rev. Elsevier; 2013;12(10):976–89.

3.	Vieth R. Why the minimum desirable serum 25-hydroxyvitamin D level should be 75 nmol/L (30 ng/ml). Best Pract Res Clin Endocrinol Metab. 2011 Aug;25(4):681–91.

4.	Baeke F, Takiishi T, Korf H, Gysemans C, Mathieu C. Vitamin D: modulator of the immune system. Curr Opin Pharmacol. Elsevier; 2010;10(4):482–96.

5.	Khajehzadeh I, Vale B. How New Zealanders distribute their daily time between home indoors, home outdoors and out of home. Kōtuitui New Zeal J Soc Sci Online. Taylor & Francis; 2017;12(1):17–31.

6.	Klepeis NE, Nelson WC, Ott WR, Robinson JP, Tsang AM, Switzer P, et al. The National Human Activity Pattern Survey (NHAPS): a resource for assessing exposure to environmental pollutants. J Expo Sci Environ Epidemiol. Nature Publishing Group; 2001;11(3):231–52.

7.	Matz CJ, Stieb DM, Davis K, Egyed M, Rose A, Chou B, et al. Effects of age, season, gender and urban-rural status on time-activity: Canadian Human Activity Pattern Survey 2 (CHAPS 2). Int J Environ Res Public Health. Multidisciplinary Digital Publishing Institute; 2014;11(2):2108–24.

8.	Prentice A. Vitamin D deficiency: a global perspective. Nutr Rev. Oxford University Press Oxford, UK; 2008;66(suppl_2):S153–64.

9.	Webb AR. Who, what, where and when—influences on cutaneous vitamin D synthesis. Prog Biophys Mol Biol. 2006;92(1):17–25.

10.	Lamberg-Allardt C. Vitamin D in foods and as supplements. Prog Biophys Mol Biol. 2006;92(1):33–8.

11.	Troesch B, Hoeft B, McBurney M, Eggersdorfer M, Weber P. Dietary surveys indicate vitamin intakes below recommendations are common in representative Western countries. Br J Nutr. 2012/06/13. Cambridge University Press; 2012;108(4):692–8.

12.	Huiberts LM, Smolders KCHJ. Effects of vitamin D on mood and sleep in the healthy population: interpretations from the serotonergic pathway. Sleep Med Rev. Elsevier; 2021;55:101379.

13.	Patrick RP, Ames BN. Vitamin D and the omega-3 fatty acids control serotonin synthesis and action, part 2: relevance for ADHD, bipolar disorder, schizophrenia, and impulsive behavior. FASEB J Off Publ Fed Am Soc Exp Biol. United States; 2015 Jun;29(6):2207–22.

14.	Patrick RP, Ames BN. Vitamin D hormone regulates serotonin synthesis. Part 1: relevance for autism. FASEB J. Federation of American Societies for Experimental Biology Bethesda, MD, USA; 2014;28(6):2398–413.

15.	Grant WB, Whiting SJ, Schwalfenberg GK, Genuis SJ, Kimball SM. Estimated economic benefit of increasing 25-hydroxyvitamin D concentrations of Canadians to or above 100 nmol/L. Dermatoendocrinol. Taylor & Francis; 2016;8(1):e1248324.

16.	Holick MF. Sunlight, UV radiation, vitamin d, and skin cancer: how much sunlight do we need? In: Sunlight, Vitamin D and Skin Cancer. Springer; 2020. p. 19–36.

17.	de Gruijl FR, Pavel S. The effects of a mid-winter 8-week course of sub-sunburn sunbed exposures on tanning, vitamin D status and colds. Photochem Photobiol Sci. Royal Society of Chemistry; 2012;11(12):1848–54.

18.	Tuckey RC, Cheng CYS, Slominski AT. The serum vitamin D metabolome: what we know and what is still to discover. J Steroid Biochem Mol Biol. Elsevier; 2018;186:4–21.

19.	Bogh MKB, Gullstrand J, Svensson A, Ljunggren B, Dorkhan M. Narrowband ultraviolet B three times per week is more effective in treating vitamin D deficiency than 1600 IU oral vitamin D3 per day: a randomized clinical trial. Br J Dermatol. Wiley Online Library; 2012;167(3):625–30.

20.	Biersack MG, Hajdukiewicz M, Uebelhack R, Franke L, Piazena H, Klaus P, et al. Sustained Increase of 25-Hydroxyvitamin D Levels in Healthy Young Women during Wintertime after Three Suberythemal UV Irradiations-The MUVY Pilot Study. PLoS One. 2016;11(7):e0159040.

21.	Grigalavicius M, Moan J, Dahlback A, Juzeniene A. Vitamin D and ultraviolet phototherapy in Caucasians. J Photochem Photobiol B Biol. Elsevier; 2015;147:69–74.

22.	Jager N, Schoepe J, Wagenpfeil S, Bocionek P, Saternus R, Vogt T, et al. The impact of UV-dose, body surface area exposed and other factors on cutaneous vitamin D synthesis measured as serum 25 (OH) D concentration: systematic review and meta-analysis. Anticancer Res. International Institute of Anticancer Research; 2018;38(2):1165–71.

23.	Orlova T, Moan J, Lagunova Z, Aksnes L, Terenetskaya I, Juzeniene A. Increase in serum 25-hydroxyvitamin-D3 in humans after sunbed exposures compared to previtamin D3 synthesis in vitro. J Photochem Photobiol B Biol. Elsevier; 2013;122:32–6.

24.	Bogh MKB, Schmedes A V, Philipsen PA, Thieden E, Wulf HC. A small suberythemal ultraviolet B dose every second week is sufficient to maintain summer vitamin D levels: a randomized controlled trial. Br J Dermatol. Wiley Online Library; 2012;166(2):430–3.

25.	Vähävihu K, Ylianttila L, Kautiainen H, Viljakainen H, Lamberg‐Allardt C, Hasan T, et al. Narrowband ultraviolet B course improves vitamin D balance in women in winter. Br J Dermatol. Wiley Online Library; 2010;162(4):848–53.

26.	IEC62471. Photobiological Safety of Lamps and Lamp Systems. Geneva, Switzerland: International Electrotechnical Commission; 2006.

27.	Diffey BL. Modelling the seasonal variation of vitamin D due to sun exposure. Br J Dermatol. Wiley Online Library; 2010;162(6):1342–8.

28.	Hyppönen E, Power C. Hypovitaminosis D in British adults at age 45 y: nationwide cohort study of dietary and lifestyle predictors. Am J Clin Nutr. Oxford University Press; 2007;85(3):860–8.

29.	French CB, McDonnell SL, Vieth R. 25-Hydroxyvitamin D variability within-person due to diurnal rhythm and illness: a case report. J Med Case Rep. BioMed Central; 2019;13(1):29.

30.	Jones KS, Redmond J, Fulford AJ, Jarjou L, Zhou B, Prentice A, et al. Diurnal rhythms of vitamin D binding protein and total and free vitamin D metabolites. J Steroid Biochem Mol Biol. 2017;172:130–5.

31.	Carney CE, Buysse DJ, Ancoli-Israel S, Edinger JD, Krystal AD, Lichstein KL, et al. The consensus sleep diary: standardizing prospective sleep self-monitoring. Sleep. Oxford University Press; 2012;35(2):287–302.

32.	Roenneberg T, Wirz-Justice A, Merrow M. Life between clocks: daily temporal patterns of human chronotypes. J Biol Rhythms. Sage Publications; 2003;18(1):80–90.

33.	Beurskens AJHM, Bültmann U, Kant Ij, Vercoulen JHMM, Bleijenberg G, Swaen GMH. Fatigue among working people: validity of a questionnaire measure. Occup Environ Med. BMJ Publishing Group Ltd; 2000;57(5):353–7.

34.	Hedlund L, Brekke HK, Brembeck P, Augustin H. A short questionnaire for assessment of dietary vitamin D intake. Eur J Nutr Food Saf. 2014;150–6.

35.	Bogh MKB, Schmedes A V, Philipsen PA, Thieden E, Wulf HC. Interdependence between body surface area and ultraviolet B dose in vitamin D production: a randomized controlled trial. Br J Dermatol. Wiley Online Library; 2011;164(1):163–9.

36.	Libon F, COURTOIS J, Le Goff C, Lukas P, Fabregat Cabello N, Seidel L, et al. Effect of body site and surface on vitamin D and 25-hydroxyvitamin D production after a single narrowband UVB exposure. J Invest Dermatol. Elsevier BV; 2017;137(6):1391–3.

37.	Muscogiuri G, Barrea L, Scannapieco M, Di Somma C, Scacchi M, Aimaretti G, et al. The lullaby of the sun: the role of vitamin D in sleep disturbance. Sleep Med. Netherlands; 2019 Feb;54:262–5.

38.	Roy S, Sherman A, Monari-Sparks MJ, Schweiker O, Hunter K. Correction of low vitamin D improves fatigue: effect of correction of low vitamin D in fatigue study (EViDiF Study). N Am J Med Sci. Wolters Kluwer--Medknow Publications; 2014;6(8):396.

39.	Nowak A, Boesch L, Andres E, Battegay E, Hornemann T, Schmid C, et al. Effect of vitamin D3 on self-perceived fatigue: A double-blind randomized placebo-controlled trial. Medicine (Baltimore). 2016;95(52).

40.	Holick MF, Chen TC. Vitamin D deficiency: a worldwide problem with health consequences. Am J Clin Nutr. Oxford University Press; 2008;87(4):1080S-1086S.
